# The mitochondrial calcium uniporter promotes arrhythmias caused by high-fat diet

**DOI:** 10.1038/s41598-021-97449-3

**Published:** 2021-09-08

**Authors:** Leroy C. Joseph, Michael V. Reyes, Edwin A. Homan, Blake Gowen, Uma Mahesh R. Avula, Chris N. Goulbourne, Elaine Y. Wan, John W. Elrod, John P. Morrow

**Affiliations:** 1 Department of Medicine, College of Physicians and Surgeons of Columbia University, New York, NY 10032 USA; 2 Center for Dementia Research, Nathan S. Kline Institute, Orangeburg, NY USA; 3 Lewis Katz School of Medicine at Temple University, 3500 N Broad St, MERB 949, Philadelphia, PA USA; 4 College of Physicians and Surgeons of Columbia University, PH10-203, 650 W 168th Street, New York, NY 10032 USA; 5 Present Address: Division of Nephrology, Department of Medicine, University of Mississippi Medical Center, Jackson, MS USA

**Keywords:** Arrhythmias, Cardiovascular biology

## Abstract

Obesity and diabetes increase the risk of arrhythmia and sudden cardiac death. However, the molecular mechanisms of arrhythmia caused by metabolic abnormalities are not well understood. We hypothesized that mitochondrial dysfunction caused by high fat diet (HFD) promotes ventricular arrhythmia. Based on our previous work showing that saturated fat causes calcium handling abnormalities in cardiomyocytes, we hypothesized that mitochondrial calcium uptake contributes to HFD-induced mitochondrial dysfunction and arrhythmic events. For experiments, we used mice with conditional cardiac-specific deletion of the mitochondrial calcium uniporter (*Mcu*), which is required for mitochondrial calcium uptake, and littermate controls. Mice were used for in vivo heart rhythm monitoring, perfused heart experiments, and isolated cardiomyocyte experiments. MCU KO mice are protected from HFD-induced long QT, inducible ventricular tachycardia, and abnormal ventricular repolarization. Abnormal repolarization may be due, at least in part, to a reduction in protein levels of voltage gated potassium channels. Furthermore, isolated cardiomyocytes from MCU KO mice exposed to saturated fat are protected from increased reactive oxygen species (ROS), mitochondrial dysfunction, and abnormal calcium handling. Activation of calmodulin-dependent protein kinase (CaMKII) corresponds with the increase in arrhythmias in vivo. Additional experiments showed that CaMKII inhibition protects cardiomyocytes from the mitochondrial dysfunction caused by saturated fat. Hearts from transgenic CaMKII inhibitor mice were protected from inducible ventricular tachycardia after HFD. These studies identify mitochondrial dysfunction caused by calcium overload as a key mechanism of arrhythmia during HFD. This work indicates that MCU and CaMKII could be therapeutic targets for arrhythmia caused by metabolic abnormalities.

## Introduction

Obese patients are at increased risk for several types of arrhythmia, including atrial fibrillation^1^. More importantly, obese patients have an increased risk of sudden cardiac death (SCD), which is often caused by ventricular tachycardias or ventricular fibrillation (VT/VF). Epidemiologic studies from several countries show that obese patients have approximately twice the risk of SCD, and diabetics have three times the risk, as age matched controls^2–5^. Although some of this may be explained by coronary artery atherosclerosis, the increased risk of SCD is greater than the increased risk of myocardial infarction in some studies, suggesting that arrhythmic events are increased more than coronary events in obese patients.

Despite the prevalence of obesity and diabetes in the general population, we have a very limited understanding of the pathophysiology that links metabolic abnormalities to arrhythmias. Obese humans have electrophysiologic abnormalities, manifest as increased frequency of ventricular ectopy (premature ventricular complexes: PVCs) and abnormal cardiac repolarization, manifest as long QT (LQT)^6–8^. LQT is an independent risk factor for cardiovascular mortality^9,10^. Humans with obesity or diabetes also have increased repolarization dispersion^11–13^, a measure of repolarization heterogeneity. QT dispersion predicts sudden cardiac death in the general population^14–16^. Weight loss surgery decreases both QT and QT dispersion, indicating that there is a causal link between obesity and repolarization abnormalities^17^. Excessive deposition of lipids in cardiac tissue is postulated to have a causal role in the pathophysiology^18^.

The composition of dietary fats is associated with variation in ECG parameters including QT interval^19^. Furthermore, clinical studies have shown that higher levels of serum fatty acids and higher dietary saturated fat intake predict SCD^20–23^, suggesting that the effects of saturated fat on the heart may be more important than obesity per se. Given the amount of saturated fat in a typical Western diet, the harmful consequences of dietary saturated fat are of general interest to society.

Mitochondrial dysfunction is thought to be an important component of the pathophysiology of heart disease caused by obesity and diabetes^24,25^. Our prior work showed that high fat diet (HFD) activates NADPH oxidase 2 (NOX2) in the heart, which increases oxidative stress and causes calcium handling abnormalities^26^. We hypothesized that mitochondrial dysfunction due to mitochondrial calcium overload has a causal role in arrhythmia during high-fat diet, upstream of repolarization abnormalities. The mitochondrial calcium uniporter (MCU, gene *Mcu*) is an important regulator of physiologic mitochondrial function. However, in times of metabolic stress, MCU could contribute to pathology. For example, increases in mitochondrial calcium, mediated by MCU, have been shown to cause cardiomyocyte death after myocardial infarction^27^.

## Results

### High fat diet causes prolonged repolarization in control but not mitochondrial calcium uniporter (MCU) KO mice

**Figure 1:**
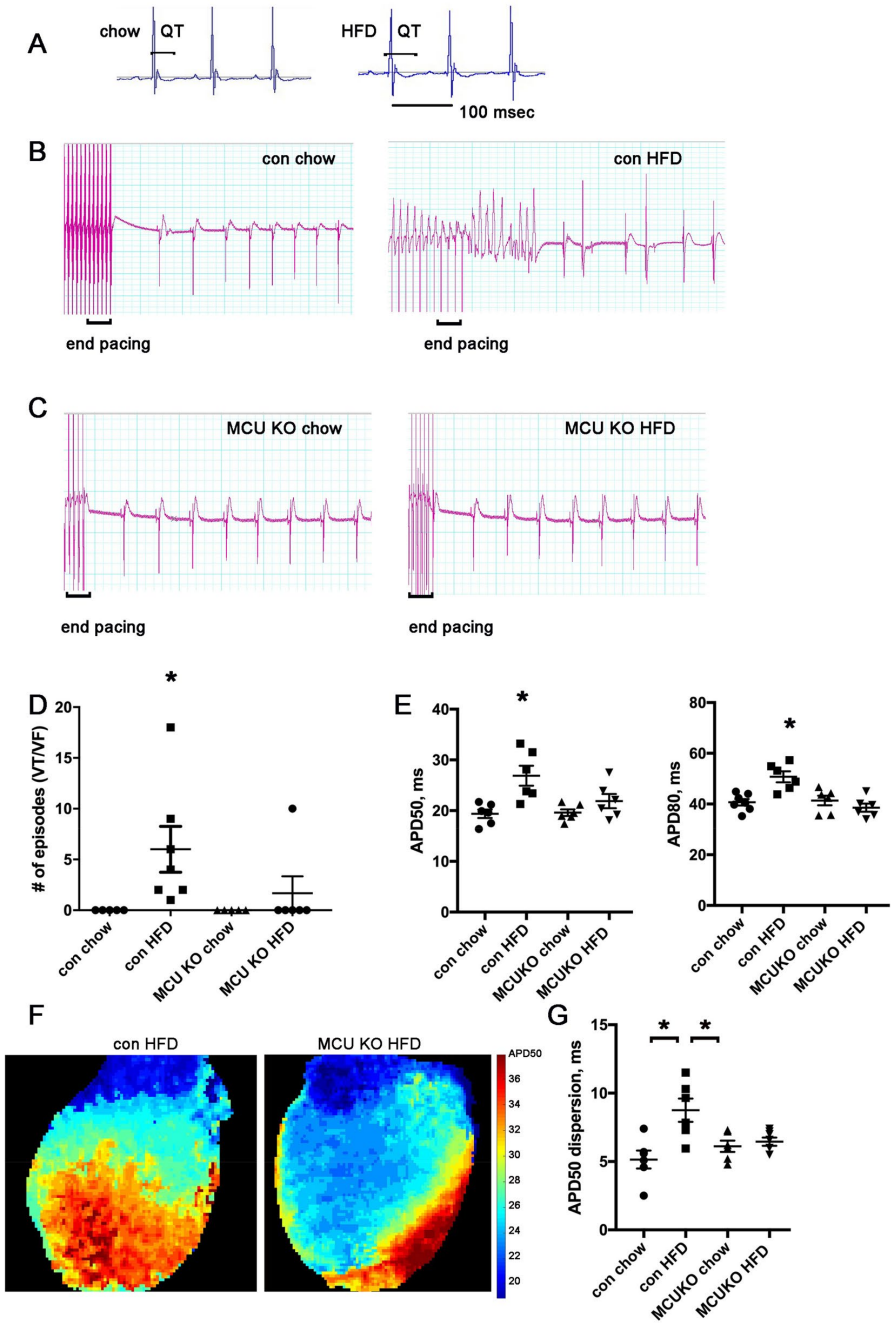
MCU KO hearts are protected from ventricular tachycardia and repolarization dispersion after HFD. (**A**) Examples of telemetry from control mouse, with QT at baseline on chow and QT prolongation after 4 weeks of HFD. Both images have the same time scale. (**B**) Two examples of heart rhythm from control hearts on chow or HFD, showing rapid ventricular pacing (at different rates) followed by non-sustained VT in the HFD heart. Time scale: each vertical thick line is 500 ms, each thin line is 100 ms. (**C**) Two examples of heart rhythm from MCU KO heart on chow or HFD, showing that rapid ventricular pacing does not cause VT. Time scale is the same as panel (**B**). (**D**) Graph of number of episodes of VT from control and MCU hearts induced by rapid ventricular pacing. N = hearts 5–7 per group. The differences between groups are statistically significant by nonparametric Kruskal–Wallis test, p = 0.0018, *indicates significantly different by post-hoc test. E. Graphs of action potential duration 50% (APD50) and 80% (APD80). The means are significantly different by ANOVA, *indicates significantly different from control by post-hoc test. F. Examples of optical mapping showing APD50 duration, both hearts were paced from the ventricular apex at 10 Hz. False color shows the gradient of APD50 from 20 ms (dark blue) to 40 ms (dark red). Note the increased repolarization dispersion in the control HFD heart compared to MCU KO HFD. G. Graph of APD dispersion in milliseconds, mean + SEM, n = 6–7 hearts per group. The means are significantly different by ANOVA, *indicates significantly different by post-hoc test.

Mitochondrial calcium levels are critical for normal cardiac physiology. Based on our prior work showing that saturated fat causes calcium handling abnormalities in cardiomyocytes^28^, we hypothesized that mitochondrial calcium overload would be an important component of the pathophysiology of high-fat diet induced arrhythmia. To test this idea, we fed cardiac specific, inducible MCU KO mice and control littermates a high fat diet for 4 weeks, after surgically implanting heart rhythm telemeters. This short duration of HFD did not cause obesity (Supplement Table 1). Telemetry recordings demonstrated that the HFD caused a prolongation in the QT intervals in control animals, indicating abnormal repolarization (Fig. 1A and Table 1). Cardiac MCU KO mice did not develop abnormal repolarization on HFD (Table 1). None of the other electrocardiographic intervals showed any significant changes. We did not detect spontaneous arrhythmias in these mice. We also determined that HFD did not cause any significant change in MCU protein level in the heart (Supplement Fig. 1).

**Table 1:** QT prolongs with HFD diet in control mice but not MCU KO mice.

	RR	PR	QRS	QT
**Control**				
Basal	101.3 ± 1.7	31.4 ± 0.8	12.0 ± 0.1	43.8 ± 0.6
HFD 4 wks	103.3 ± 1.0	33.1 ± 0.7	12.3 ± 0.1	55.9 ± 4.0*
**MCU KO**				
Basal	105.1 ± 1.8	32.4 ± 0.9	11.9 ± 0.3	44.6 ± 1.5
HFD 4 wks	104.6 ± 1.3	33.6 ± 0.8	12.1 ± 0.3	44.9 ± 1.7

Intervals are in milliseconds, ± SEM, n = 4 each group, *p < 0.05 compared to baseline.

### MCU KO hearts are protected from ventricular tachycardia after high fat diet

Next, we evaluated susceptibility to arrhythmia using perfused hearts subjected to rapid ventricular pacing. All hearts went through the same pacing protocol (Supplement Table 2). None of the hearts from mice on regular chow had inducible ventricular tachycardia (VT), in either control or MCU KO genotypes. All of the hearts from control mice on HFD had inducible VT (7 of 7). In contrast, only one of the six hearts from MCU KO mice on HFD had inducible VT, indicating that this genotype is protected from arrhythmia caused by high-fat diet (Fig. 1B–D). To achieve a better understanding of the arrhythmogenic substrate, we performed optical mapping on perfused hearts with a voltage-sensitive dye. Control hearts had a significant increase in mean action potential duration 50% and 80% (APD50, APD80) but there was no difference in APD with MCU KO hearts comparing chow to HFD (Fig. 1E). In addition, after high-fat diet, control hearts had significantly increased ventricular repolarization dispersion, indicating greater spatial heterogeneity. This is an established substrate for arrhythmia^29^. In contrast, MCU KO hearts did not have increased repolarization dispersion after the same duration of HFD (Fig. 1F,G). Thus, the absence of abnormal arrhythmia substrate correlates with the reduced inducibility of VT for this genotype. Conduction velocity was similar in all groups (Supplement Table 3).

### High fat diet causes activation of calmodulin-dependent protein kinase (CaMKII) in control hearts but not MCU KO hearts

**Figure 2:**
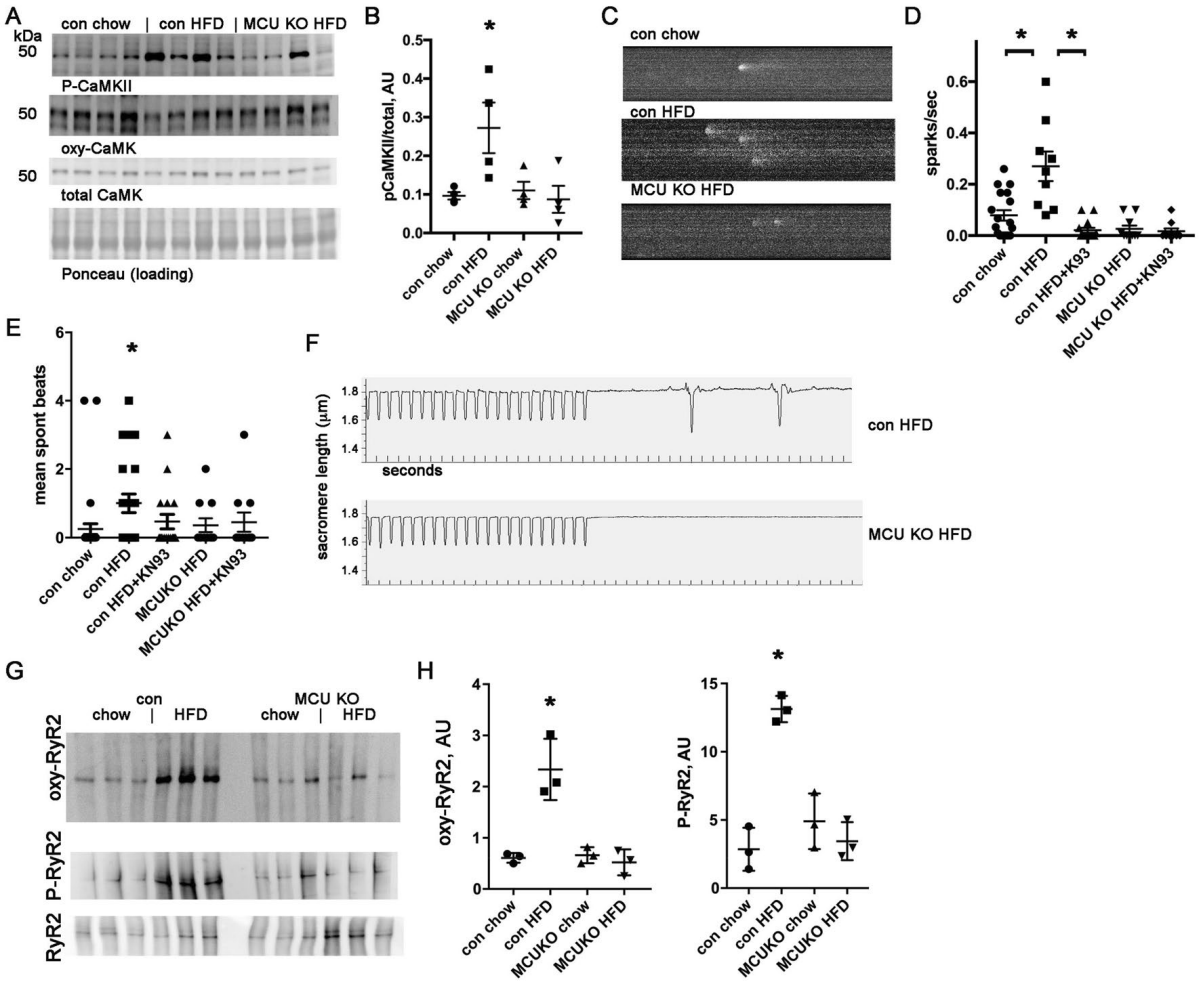
MCU KO hearts are protected from CaMKII activation caused by HFD and have fewer sparks and fewer spontaneous contractions. (**A**) Immunoblots of phospho-CaMKII, oxy-CaMKII, and total CaMKII from heart lysates. (**B**) Graph of immunoblots phospho-CaMKII corrected for total CaMKII. (**C**) Representative images of calcium sparks. (**D**) Graph of quantification of calcium sparks, n = 12–20 cardiomyocytes from 3 isolations, *indicates significantly different by post-hoc test. KN93 is a CamKII inhibitor, 0.5 µM. (**E**) Graph of quantification of spontaneous beats following pacing in cardiomyocytes. (**F**) Representative images of single cell contractility and spontaneous beats following pacing. (**G**) Representative images of oxidized RyR2, measured by DNP modification, phosphorylated RyR2 (ser2814) and total RyR2. (**H**) Graphs of quantification of oxidized RyR2 and P-RyR2 (normalized to total RyR2), both are sig different by ANOVA, *Indicates significantly different from control chow by post-hoc test.

To better understand the molecular pathways involved in the mechanisms of arrhythmia, we evaluated CaMKII, which has a prominent role in the pathophysiology of both heart failure ^30^ and arrhythmia ^31^. We found that CaMKII is activated in control hearts after HFD, but not in MCU KO hearts (Fig. 2A,B), as measured by phosphorylation of CaMKII at threonine 286. We did not detect any significant difference in oxidized CaMKII (Supplemental Fig. 2). Since abnormal SR calcium leak can promote arrhythmia, we quantified calcium sparks in cardiomyocytes isolated from mouse hearts after 4 weeks of HFD or control animals on regular chow. This showed a significantly greater number of sparks in control cardiomyocytes after HFD, an effect that was decreased by CaMKII inhibition with the selective inhibitor KN93. MCU KO cardiomyocytes did not have increased sparks after HFD, correlating with the absence of CaMKII activation in this genotype (Fig. 2C,D).

To evaluate the arrhythmogenic substrate at the single cell level, we also quantified contractions in isolated cardiomyocytes after a brief period of pacing to achieve steady-state intracellular calcium. This showed a pattern similar to the calcium sparks data: after HFD, control cardiomyocytes had a significant increase in contractions, which is decreased by CaMKII inhibition (Fig. 2E,F). MCU KO cardiomyocytes did not have an increase in contractions after HFD compared to control cardiomyocytes. Post-translational modification of RyR2 channels is involved in several forms of heart disease and contributes to abnormal intracellular calcium handling. RyR2 is both oxidized and phosphorylated in control ventricles after HFD (Fig. 2G,H). This could account for the increase in calcium sparks seen in control cardiomyocytes after HFD. We detected minimal changes in RyR2 from the MCU KO ventricles after HFD, indicating that mitochondrial dysfunction from calcium overload is necessary for these post-translational modifications of RyR2 (Fig. 2G,H).

### High fat diet reduces Kv1.5 protein levels in control hearts but not in MCU KO hearts

**Figure 3:**
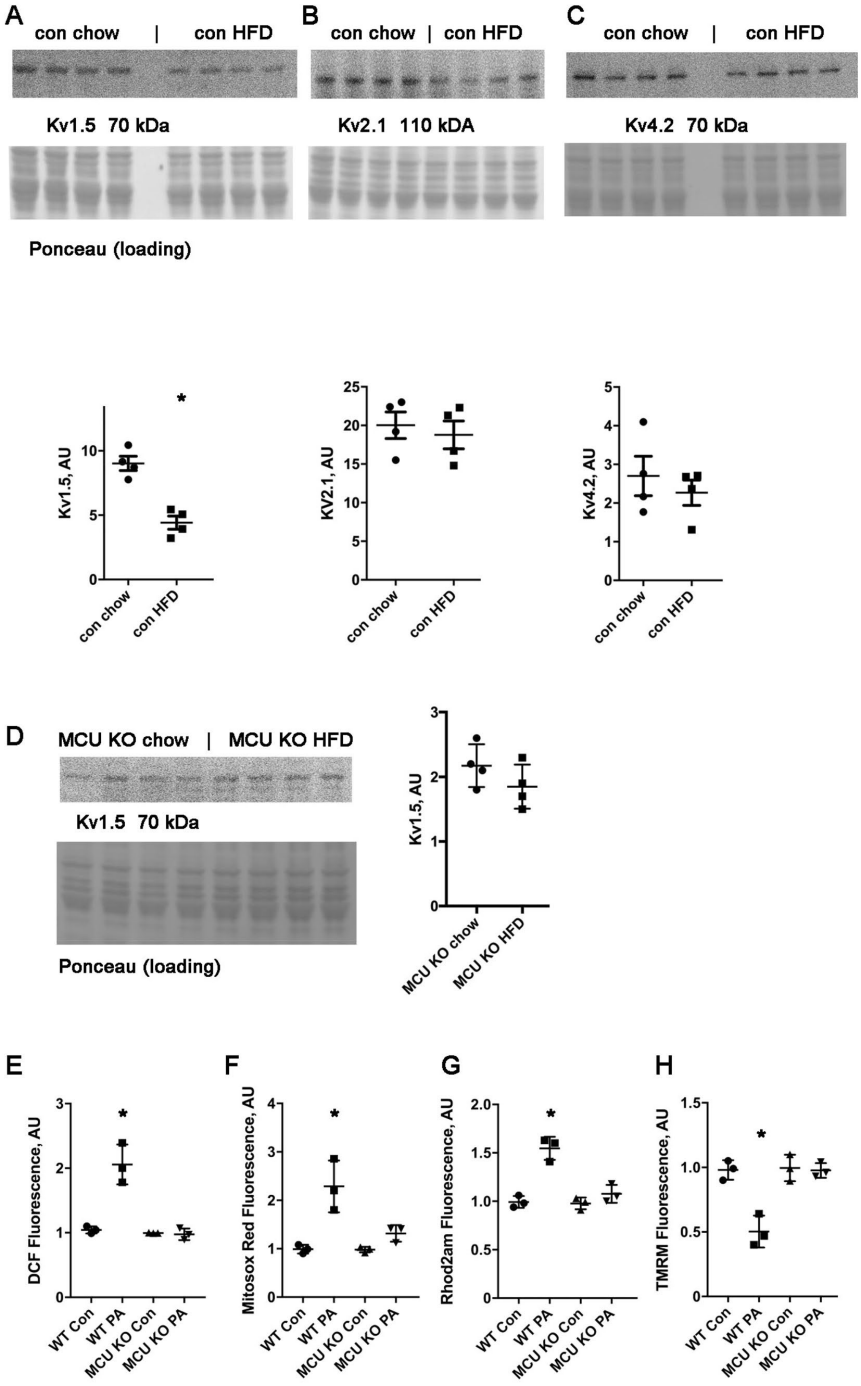
Kv1.5 protein levels are reduced in control hearts on HFD; MCU KO cardiomyocytes are protected from ROS and mitochondrial dysfunction induced by saturated fat. (**A**) Western blot and graph of Kv1.5 from control hearts, *Indicates sig different from con chow by t-test. Graph values normalized to loading control, n = 4 heart lysates per group. (**B**) Western blot and graph of Kv2.1 from control hearts. (**C**) Western blot and graph of Kv4.2 from control hearts. (**D**) Western blot and graph of Kv1.5 from MCU KO hearts. (**E**) Isolated control and MCU KO cardiomyocytes, ± palmitate, height is DCF fluorescence minus background, mean + SEM, n = triplicate wells, 2000 cells per well from one heart for each genotype. (**F**) Control and MCU KO cardiomyocytes, mitosox red signal. (**G**) Control and MCU KO cardiomyocytes, Rhod2AM signal for mitochondrial calcium. Values for each genotype was normalized to the corresponding control condition for that genotype. (**H**) Control and MCU KO cardiomyocytes, TMRM signal to indicate mitochondrial inner membrane potential. For all graphs shown, the means are significantly different by ANOVA, *Indicates significantly different from control by post-hoc test. PA = palmitate 200 uM 4 h. For (**E**–**H**), similar results were obtained from 3 different isolations for each genotype.

To investigate the cause of abnormal repolarization, we performed western blots of ventricular tissue lysates for several voltage-gated potassium channels. We found that there was a significant reduction in Kv1.5 protein levels. There was no significant difference in Kv2.1 or Kv4.2 protein levels (Fig. 3A–C). A reduction in voltage-gated potassium channels would be expected to cause an increase in APD. In contrast to control heart lysates, MCU KO hearts had a mild, nonsignificant decrease in Kv1.5 protein levels (Fig. 3D).

### MCU KO cardiomyocytes are protected from ROS and mitochondrial dysfunction induced by saturated fat

To evaluate the cellular abnormalities caused by saturated fat in more detail, we performed experiments with isolated cardiomyocytes. Consistent with our prior work, control cardiomyocytes show a significant increase in total ROS and mitochondrial ROS after exposure to the saturated fatty acid palmitate, bound to BSA. MCU KO cardiomyocytes do not have an increase in ROS after exposure to palmitate (Fig. 3E,F). Control cardiomyocytes also have a significant increase in mitochondrial calcium content and a decrease in inner membrane potential after exposure to palmitate, indicating mitochondrial dysfunction. MCU KO cardiomyocytes are also protected from these abnormalities (Fig. 3G,H).

### CaMKII inhibition protects cardiomyocytes from mitochondrial dysfunction induced by saturated fatty acids and protects from ventricular tachycardia after HFD

**Figure 4:**
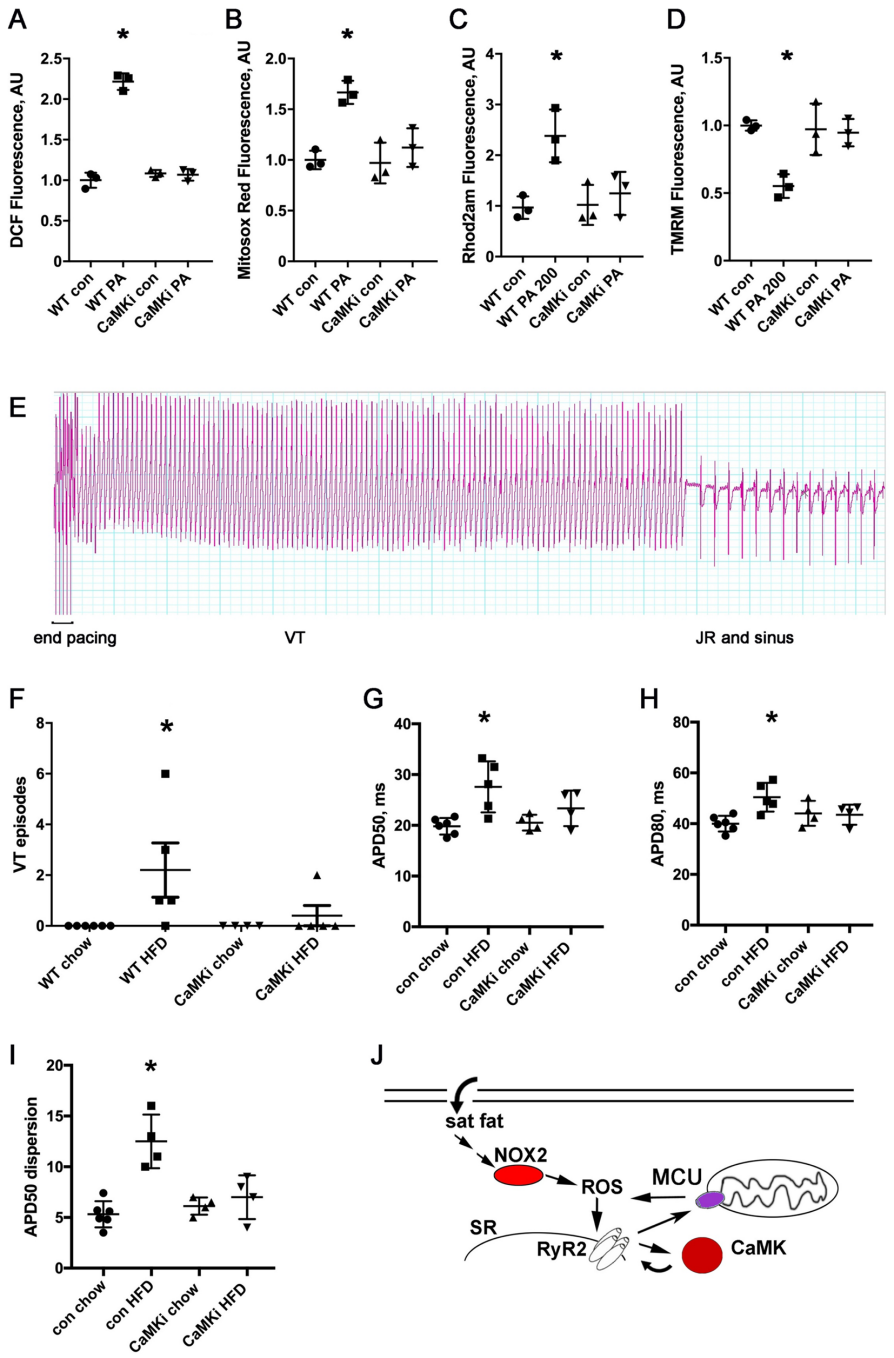
CaMK inhibitor cardiomyocytes are protected from ROS and mitochondrial dysfunction, and CaMK inhibitor hearts are protected from ventricular tachycardia and repolarization dispersion after HFD. (**A**) Isolated WT and CaMKi transgenic cardiomyocytes, ± palmitate, height is DCF fluorescence minus background, mean + SEM, n = triplicate wells, 2000 cells per well. (**B**) WT and CaMKi transgenic cardiomyocytes, mitosox red signal. (**C**) WT and CaMKi transgenic cardiomyocytes, Rhod2AM signal for mitochondrial calcium. (**D**) WT and CaMKi transgenic cardiomyocytes, TMRM signal to indicate mitochondrial inner membrane potential. For (**A**–**D**), similar results were obtained from 3 different isolations for each genotype. (**E**) Example of a longer episode of burst-pacing induced VT from a WT HFD heart, followed by several beats of junction rhythm (JR, note the absence of p-waves) and then sinus rhythm resumes. Time scale: each vertical thick line is 500 ms, each thin line is 100 ms. (**F**) Graph of number of episodes of VT from control and CaMKi hearts induced by rapid ventricular pacing. N = 4–6 hearts per group. The differences between groups are statistically significant by nonparametric Kruskal–Wallis test, p = 0.014. (**G**) Graph of action potential duration 50% (APD50). The means are significantly different by ANOVA, *Indicates significantly different from control by post-hoc test. (**H**) Graph of action potential duration 80% (APD80). The means are significantly different by ANOVA, *Indicates significantly different from control by post-hoc test. (**I**) Graph of APD dispersion, n = 4–6 hearts per group, the means are significantly different by ANOVA, *Indicates significantly different by post-hoc test. (**J**) Schematic of proposed mechanism. Saturated fatty acid increases oxidative stress in cardiomyocytes by activating NOX. This causes increase calcium leak from the sarcoplasmic reticulum, leading to mitochondrial calcium overload and mitochondrial dysfunction. Preventing mitochondrial calcium overload is protective. This pathophysiology is exacerbated by CaMK activation. *MCU* mitochondrial calcium uniporter, *NOX2* NADPH oxidase 2, *ROS* reactive oxygen species, *RyR2* ryanodine receptor 2, *SR* sarcoplasmic reticulum.

To understand the role of CaMKII in the mitochondrial dysfunction caused by saturated fat, we used transgenic mice that express a peptide inhibitor of CaMKII in the heart (CaMKi)^30^. We performed experiments with isolated cardiomyocytes from CaMKi hearts, compared to isolated cardiomyocytes from WT littermates. CaMKII inhibition essentially normalized total and mitochondrial ROS (Fig. 4A,B). Measurements of mitochondrial calcium and inner membrane potential also showed a strong protective effect of CaMK inhibition (Fig. 4C,D). This demonstrates that CaMKII has an important role in the increase in ROS and mitochondrial dysfunction caused by saturated fatty acids in cardiomyocytes.

We also used these transgenic CaMKi mice to examine the role of CaMK in intact hearts. We fed groups of these mice and their WT littermates the high-fat diet for 4 weeks. VT was not induced in hearts from animals on chow. VT was induced in the majority of the WT hearts after HFD (4/5 hearts, Fig. 4E). Hearts from CaMKi mice were protected from VT after HFD: 4 out of 5 hearts did not have inducible VT (Fig. 4F). Optical mapping showed that the control littermate hearts on HFD had a significant increase in mean APD50 and APD80 compared to chow, indicating delayed ventricular repolarization, but there was no difference in mean APD50 or APD80 with CaMKi hearts (Fig. 4G,H). Optical mapping also showed that hearts from CaMKi mice did not have an increase in repolarization dispersion after HFD, similar to the beneficial effect of the MCU KO (Fig. 4I). We conclude that CaMKII inhibition offers protection from inducible VT, in part by preventing repolarization abnormalities after HFD.

We also examined MCU protein levels in the hearts of CaMKi mice compared to controls. We found, unexpectedly, that the CaMKi hearts have approximately half the MCU protein level of the hearts from control littermates, with no differences due to diet (Supplemental Fig. 3). This could also contribute to the protective effect of the CaMKi genotype.

## Discussion

The heart must beat rhythmically to sustain life. This results in a constant, intense metabolic demand on the myocardium to produce mechanical work. There is a growing appreciation that conserved molecular pathways link heart rhythm to cardiac metabolism^24,32^. Our current work provides new insights into the pathophysiology of arrhythmias caused by abnormal cardiac metabolism. We have previously shown that high-fat diet activates NOX2 in the heart, increasing oxidative stress, which promotes calcium handling abnormalities and arrhythmia^26^. We now show that the presence of MCU promotes VT during high fat diet. Cardiomyocyte deletion of MCU, the major protein responsible for rapid mitochondrial calcium uptake, is protective in this rodent model. MCU has a physiologic role in regulating mitochondrial function in response to exercise^33^ but it can contribute to pathophysiology in certain disease states. We show that MCU KO hearts have significantly less inducible VT after HFD, which is explained by the fact that these hearts have less repolarization dispersion and less intracellular calcium handling abnormalities at the cellular level. The calcium handling abnormalities and mitochondrial abnormalities are at least in part dependent on activation of CaMKII (schematic, Fig. 4J). Our data also show that inhibiting CaMKII is beneficial during HFD, since it prevents abnormal repolarization and results in protection from inducible VT in transgenic CaMKi hearts. A decrease in MCU protein may be part of the protective mechanism in this transgenic line.

Every diet contains a complex mix of nutrients and it can be challenging to determine which component is responsible for a biologic effect. Based on the isolated cardiomyocyte experiments, the saturated fatty acid palmitate is most likely the cause of the cardiac pathophysiology in this model. In addition, our prior work showed that a HFD from olive oil (which is mostly composed of monounsaturated fat) did not cause electrophysiologic changes in vivo^26^. Thus, the type of fat in the diet is probably more important than the overall fat content.

Although it is well established that mitochondria are a major source of ATP in cells, there is increasing interest in the role of mitochondria in oxidative stress and calcium handling. The pathophysiologic significance of mitochondrial calcium in heart disease is controversial. Another group has reported that mitochondrial calcium is decreased in a guinea pig heart failure model^34^, whereas others have found mitochondrial calcium overload in a mouse models of cardiomyopathy^35,36^. The use of different species and different disease models may account for the discordant results. Our work indicates that MCU is an important contributor to arrhythmia in the context of chronic metabolic stress caused by high-fat diet. Notably, the electrophysiologic abnormalities occur before the onset of obesity in this high-fat diet animal model. This work is consistent with prior reports that mitochondrial antioxidants can reduce arrhythmias in transgenic models of cardiac lipid overload^37^ and a guinea-pig heart failure model^38^, although those two animal models have more severe heart rhythm abnormalities than those present in this report. The data presented here indicate the mitochondrial calcium overload causes an increase in cardiac ROS after exposure to saturated fat. Our data also indicate that CaMKII has a role in the mitochondrial dysfunction induced by saturated fat since transgenic cardiac CaMK inhibitor mice are also protected. There is controversy regarding the ability of CaMKII to regulate MCU directly in cardiac myocytes^39,40^. It is not necessary to invoke this mechanism to explain our findings. Even if MCU activity is unchanged from baseline, chronic SR calcium leak could lead to mitochondrial calcium overload.

Repolarization abnormalities appear to play a role in the pathophysiology of arrhythmia after HFD. Our work indicates that HFD can cause a reduction in Kv1.5, which produces a repolarizing current. This is consistent with prior work using diet-induced obese mice^41^. Prolonged repolarization and repolarization dispersion, manifest as QT dispersion on ECGs, has been shown to predict adverse events in many clinical studies^14,16^. However, the molecular mechanisms of repolarization dispersion have been elusive. Computational models have implicated both repolarization and cell–cell coupling^29^. The causes of abnormal repolarization during metabolic stress are probably complex and multi-factorial; it may be relevant that metabolic stress can activate K_ATP_ channels, resulting in abnormal repolarization^42^. In addition, CaMKII activation has complex effects on cardiac ion channels, which could contribute to abnormal repolarization.

These results from a short duration of HFD should not be extrapolated to diabetes or obesity. The molecular mechanisms involved in the cardiac pathophysiology of diabetes or obesity could be quite different and future work could focus on these comparisons.

## Study limitations

This project uses mice as a model organism. Although rodent cardiac physiology differs from human cardiac physiology in several respects, mice are an appropriate animal model for this project, which involves manipulation of dietary fat content, because they are omnivores, as are humans. This is in contrast to other animal models commonly used in cardiac research, such as guinea pigs and rabbits (herbivores) and dogs (carnivores). We have previously shown that WT diet-induced obese mice have heart rhythm abnormalities similar to obese humans, validating this model^41^. For some of our experiments, we exposed isolated cardiomyocytes to palmitate; this model system may not recapitulate the in vivo response to HFD. In addition, the fluorescent dyes used in the isolated cardiomyocytes do not allow precise measurements of absolute value and are best understood as difference in arbitrary units.

Several aspects of the cellular pathophysiology could be examined in more detail in the future. Although extracellular calcium enters cardiomyocytes with every heart-beat, it is unclear if the cytosolic calcium level is elevated with HFD, causing mitochondrial dysfunction. More likely, an increase in SR calcium leak, caused by NOX2 activation, is responsible for the increase in mitochondrial calcium. It has been proposed that the mitochondria and SR form a microdomain that allows for calcium transfer^43^.

It is likely that the mitochondrial permeability transition pore (MPTP) is involved in the reduction of the inner membrane potential. Although prolonged opening of the MPTP can cause oxidative stress and cell death, the MPTP has a physiologic role in regulating mitochondrial calcium^44^. However, increased opening of the MPTP would decrease mitochondrial calcium content, whereas we observed increased mitochondrial calcium content with decreased inner membrane potential. This could be consistent with sub-maximal activation of the MPTP. It is also possible that other proteins are involved, such as mitochondrial uncoupling proteins (UCP). The observed partial decrease in inner membrane potential would still allow for a voltage gradient that could serve as a driving force for cations to enter the mitochondria.

## Conclusions

Our work indicates that MCU, the major protein responsible for rapid uptake of mitochondrial calcium, has an important role in the pathophysiology of ventricular arrhythmia and repolarization abnormalities after high-fat diet. Deletion of MCU prevents ventricular arrhythmias. CaMKII is activated in the heart by high-fat diet, and CaMKII inhibition prevents mitochondrial dysfunction caused by saturated fat. More research will be needed to decipher the pathways connecting mitochondrial dysfunction and abnormal repolarization.

## Materials and methods

### Materials and Western blots

The OxyBlot Protein Oxidation Detection Kit and KN93 were purchased from Sigma (S7150). The phospho-CaMK antibody was purchased from Cell Signaling (12716); oxy-CaMK and total CaMKII antibodies were purchased from Genetex (GTX36254 and GTX111401). Kv channel antibodies were purchased from Alomone (APC-004, APC-012, APC-023). The MCU antibody was purchased from Cell Signaling (14997). RyR2 antibody was purchased from Thermofisher (MA3-916) and these blots were performed as previously described^45^. To make protein lysates for western blots, cardiac samples were lysed in RIPA (Thermo Scientific RIPA Lysis and Extraction Buffer) with protease and phosphatase inhibitors (Halt Protease Inhibitor Cocktails, Halt Phosphatase Inhibitor Cocktail) using a bead beater. After the addition of Laemmli sample buffer with beta-mercaptoethanol, lysates were boiled for 5 min. Chemiluminescence (West Femto Maximum Sensitivity Substrate, ThermoFisher) was recorded with a digital gel imager and quantified with ImageJ software version 1.46r (https://imagej.nih.gov/ij/download.html).

### Animal care and cardiomyocyte isolation

Wild type (WT) C57BL6J mice were purchased from Jackson Labs. The cardiac-specific inducible MCU KO mice were provided by John W. Elrod, PhD at Temple University. We bred mice that were homozygous floxed at the MCU gene, half of which also carried the tamoxifen-inducible Cre recombinase driven by the alpha myosin-heavy chain promoter^33^. Mice without Cre served as controls. Both genotypes of mice (MCU flox/flox with and without Cre) received intraperitoneal injections of 1 mg tamoxifen (Sigma, T5648) for five consecutive days and recovered for one week prior to experiments. The cardiac-transgenic CaMKII inhibitor (AC3-I) mice were transferred to us by Mark E. Anderson, MD/PhD at Johns Hopkins University^30^. All of these strains had been back-crossed to the C57BL6J background for at least 6 generations. Littermates were used as controls unless otherwise noted. Mice were used for experiments at 12–16 weeks of age. The high fat diet was a special order from Research Diets Inc: D04051707, with 60 kcal% from palm oil, which is high in saturated fat. Control chow was Picolab Rodent diet 20 from LabDiet, which has 13% kcal from fat. Animals were fed ad libitum. Isolation of cardiomyocytes was performed as previously described, using Liberase TM (Sigma)^46^. Cells were counted using 2 µl of cell suspension and a conventional light microscope. Animals were randomly assigned to treatment groups. No animals were excluded from the analysis. Optical mapping quantification were performed by blinded operators.

### Heart rhythm telemetry

Telemetry devices (Data Sciences International, model EA-F10) were implanted in mice under sterile conditions with inhaled isoflurane for anesthesia. The two subcutaneous leads were positioned to approximate ECG limb lead II. The mice recovered for one week after surgery before initiating recordings. ECG intervals were measured manually using Ponemah 3 software (https://www.datasci.com/docs/default-source/software/ponemah_software.pdf) from recordings with minimal artifact at the same time of day, 10am-noon. Intervals were measured manually from 5 consecutive beats by a blinded reader. Each heart rhythm recording was scanned in its entirety by a trained researcher to detect arrhythmias. Power calculation: With a low frequency of ventricular ectopy in control mice, alpha error of 5% and a predicted increase in PVCs of 50% with the high-fat diet, 4 mice per group has statistical power of 95%.

### Arrhythmia induction and optical mapping protocol

The operator was blinded to group assignment. Mice were injected with heparin prior to administration of isoflurane. Hearts were isolated and perfused via a Langendorff apparatus with warm oxygenated Krebs–Henseleit buffer (pH 7.4; 95% O_2_, 5% CO_2_, 37 °C). Susceptibility to pacing-induced VT/VF was assessed by a standardized pacing protocol (increasing from 10 to 20 Hz, see Supplemental Table 2) at twice the excitation threshold of the left ventricle, using two silver chloride electrodes spaced 2 mm apart, placed on the apex of the perfused hearts. Energy for pacing was provided by an AD instruments stimulator HC module controlled by AD Instruments LabChart v8. Our laboratory had previously tested this pacing protocol to establish that it did not induce VT/VF in control hearts. We allowed 10 s in between rounds of pacing. If a round of pacing triggered VT/VF, we waited until spontaneous termination, then proceed with the pacing protocol after waiting 10 s. VT was defined as three or more consecutive beats of ventricular origin. For optical mapping, hearts were placed in a glass chamber in a Tyrode bath for superfusion. Silver chloride electrodes were positioned near the hearts to record an ECG. Blebbistatin (5–10 µM) was perfused to reduce motion, and Di-4-ANEPPS (100 µM) was perfused to record optical membrane potentials. Hearts were paced at the ventricular apex (Pulsar 6i, FHM, Brunswick, ME). Hearts were illuminated with green excitation lasers (532 nm) to activate Di-4-ANEPPS. Emitted fluorescence was captured through a 715-nm pass filter using a complementary metal–oxide–semiconductor (CMOS) camera (MICAM Ultima, SciMedia). Movies were acquired at 1000 frames per second for a duration of 4–5 s, with 100 × 100-pixel resolution (0.095 mm per pixel). Optical movies were acquired during ventricular pacing. Perfused hearts were discarded after these experiments and were not used to make protein lysates since there could be alterations in calcium cycling or metabolism due to pacing.

### Optical mapping data processing and analysis

Recorded optical movies were processed using software developed by the Efimov laboratory^47^. The background fluorescence was subtracted from each frame, and spatial (5 × 5 pixels) and temporal (9 frames) conical convolution filters were used to increase signal-to-noise ratio. Optical mapping movies were spatio-temporarily filtered to reduce noise. Phase movies were obtained after Hilbert transformation of the fluorescent signal and APD maps were generated as previously reported^48^. APD dispersion (APD_max_ –APD_min_) was calculated from the area of the LV free wall.

### Cardiomyocyte contractility and calcium spark recording and analysis

Cardiomyocyte isolations were performed as previously described^49^. Isolated cardiomyocyte contractility was measured using an Ionoptix system as previously described^49^. Spontaneous contractions were measured for 40 s after a brief pacing interval. Sparks were recorded with a Leica SP2 confocal microscope (Wetzlar, Germany) equipped with a 63 × 1.4 NA objective. Cardiomyocytes were loaded with fluo-4 (5 μM for 10 min) in modified Tyrode solution containing 1 mM calcium. Line scan images were recorded for 10 s with and quantified with the Sparkmaster plugin of ImageJ. Isolated cardiac myocytes experiments were performed at room temperature.

### Measurements of ROS, mitochondrial calcium, and mitochondria depolarization

We used a physiologic concentration of fatty acid, (200 µM bound to bovine serum albumin (BSA, final concentration 30 µM^50^. Palmitate was used because it is one of the most abundant saturated fatty acids in the bloodstream of mammals. Cells were exposed to palmitate for four hours. After fatty acid treatment, isolated cardiomyocytes were divided into aliquots. To measure total cellular ROS, cells were loaded with the fluorescent dye 2′,7′-Dichlorofluorescin diacetate (H2DCF-DA) (25 µM). Once it enters the cell, H2DCF-DA is converted by oxidation to DCF, which is fluorescent, and this is widely used as a measure of general oxidative stress^51^. To measure mitochondrial ROS, Mitosox Red (5 µM), was used to analyze mitochondrial superoxide generation. Cells were incubated with DCF or mitosox red for 30 min in the dark. Excess DCF or Mitosox Red was removed with two washes of BSA solution. DCF fluorescence was recorded at excitation/emission wavelengths: 488/532 nm whereas MitoSOX Red was recorded at 525 (excitation) and 620 nm (emission). To assess the changes in mitochondrial membrane potential, cardiomyocytes were stained with 1 nM tetramethylrhodamine methyl (TMRM) ester for 30 min and recorded at 543 nm (excitation)/590 nm (emission). TMRM preferentially accumulates in mitochondria due to its positive charge. As mitochondria are depolarized, they trap less TMRM, and so the signal is proportional to the inner membrane potential of the mitochondria. To determine the changes in mitochondrial calcium in living cells, cardiomyocytes were loaded with 10 µM Rhod 2-AM and incubated for 30 min followed by a one-hour washout with cytosolic quenching using manganese (200uM MnCl2), to produce specific mitochondrial calcium signal^52^. Rhod2AM fluorescence was measured at 552 nm (excitation)/ 581 nm (emission). Cells were loaded onto a 96-well in triplicate, plated at 2000 cells/ well, and fluorescence was measured with a Molecular Devices iD3 plate reader.

### Statistical analysis

Results are given as mean ± SEM. The unpaired *t* test was used for comparisons of 2 means. a 2-tailed value of *P* < 0.05 was considered statistically significant. For groups of 2 or more, analysis of variance (ANOVA) was used with Tukey post hoc testing or non-parametric methods (Prism v7, GraphPad Software Inc., La Jolla, CA).

### Study approval

Animal protocols were approved by the Columbia University Institutional Animal Care and Use Committee and were carried out in accordance with the NIH guidelines for the care and use of laboratory animals as well as ARRIVE guidelines.

## Supplementary Information

Supplementary Information.
