# Supplementary material for: The mitochondrial calcium uniporter promotes arrhythmias caused by high-fat diet
Source: Sci Rep. 2021 Sep 8;11:17808. doi: 10.1038/s41598-021-97449-3 (PMC8426388; doi:10.1038/s41598-021-97449-3)

**Supplement for “The mitochondrial calcium uniporter promotes arrhythmias**

**caused by high-fat diet”**

**Supplement Table 1. Average weight change for mice on regular chow or HFD after 4 weeks.**

|  | **Weight change (g)** |
| --- | --- |
| **MCU con chow** | 1.3 ± 0.5 |
| **MCU con HFD** | 1.7 ± 0.5 |
| **MCU KO chow** | 1.3 ± 0.4 |
| **MCU KO HFD** | 1.4 ± 0.3 |
| **WT chow** | 1.5 ± 0.2 |
| **WT HFD** | 2.5 ± 0.5 |
| **CaMKi chow** | 1.6 ± 0.4 |
| **CaMKi HFD** | 2.4 ± 0.3 |

N= 10 each group. Differences between groups were not significant per ANOVA.

**Supplement Table 2. Perfused heart ventricular pacing protocol**

| Hz | Duration (beats) |  |
| --- | --- | --- |
| 10 | 20 |  |
| 12 | 20 |  |
| 14 | 20 |  |
| 16 | 20 |  |
| 18 | 20 |  |
| 20 | 20 |  |
| 10 | 40 |  |
| 12 | 40 |  |
| 14 | 40 |  |
| 16 | 40 |  |
| 18 | 40 |  |
| 20 | 40 | repeat 3x |
| 10 | 80 |  |
| 12 | 80 |  |
| 14 | 80 |  |
| 16 | 80 |  |
| 18 | 80 |  |
| 20 | 80 | repeat 3x |

**Supplement Table 3. Conduction velocity by optical mapping**

| conduction velocity by optical mapping, m/s | | |
| --- | --- | --- |
|  | mean | SD |
| con chow | 0.68 | 0.03 |
| con HFD | 0.75 | 0.13 |
| MCU KO chow | 0.73 | 0.03 |
| MCU KO HFD | 0.71 | 0.06 |
| CaMKi chow | 0.70 | 0.04 |
| CaMKi HFD | 0.69 | 0.03 |

**Supplement Figure 1: MCU western blot, control mice**

**
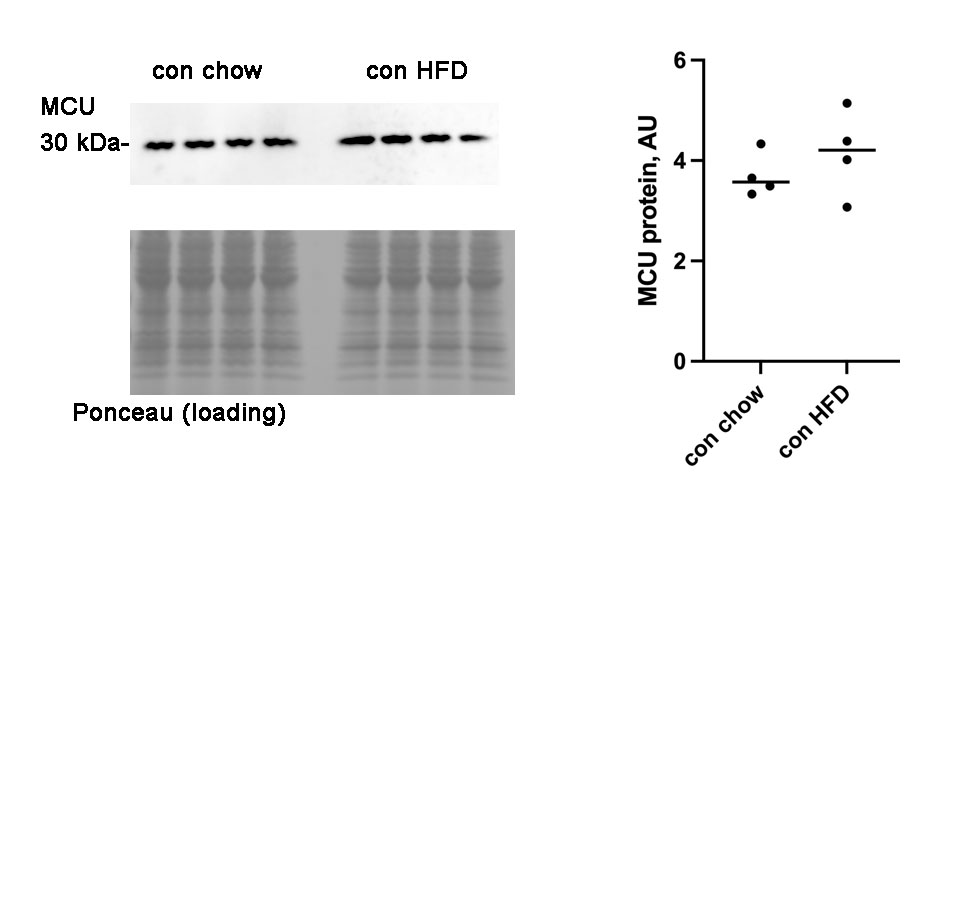
**

Shown is immunoblot from mouse hearts and Ponceau as loading control, with graph of quantification, MCU protein level adjusted for loading, p= 0.37 by t-test.

**Supplement Figure 2: Quantification of oxy-CaMK western blot**


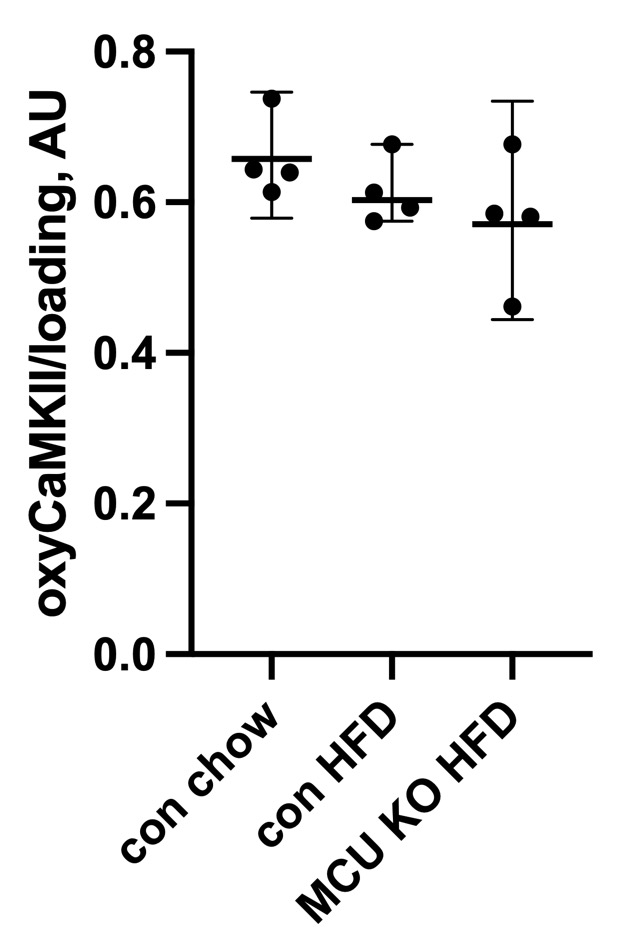


Shown is graph of quantification, immunoblot signal from mouse heart adjusted for loading, P = 0.25 by ANOVA.

**Supplement Figure 3: MCU western blot, control mice and CaMKi mice**

**
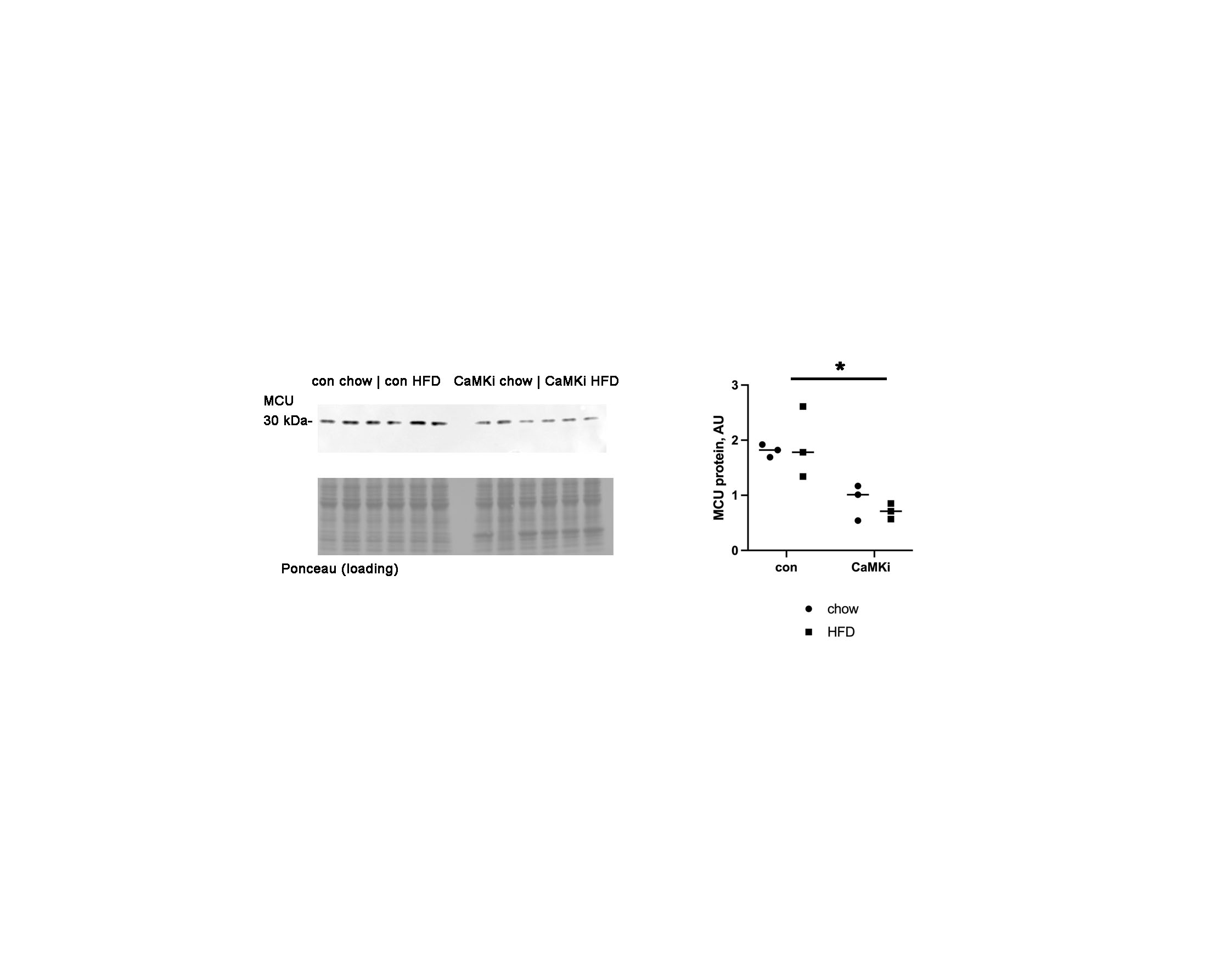
**

Shown is immunoblot from mouse hearts and Ponceau as loading control, with graph of quantification, MCU protein level adjusted for loading. 2-way ANOVA indicates the difference in genotype is significant, p= 0.0012, and there is no significant difference by diet (indicated by symbol shape).

**Supplemental material:** Full uncut gels, all images shown were obtained by the authors.

**Figure 2A:** Phospho-CaMK

**
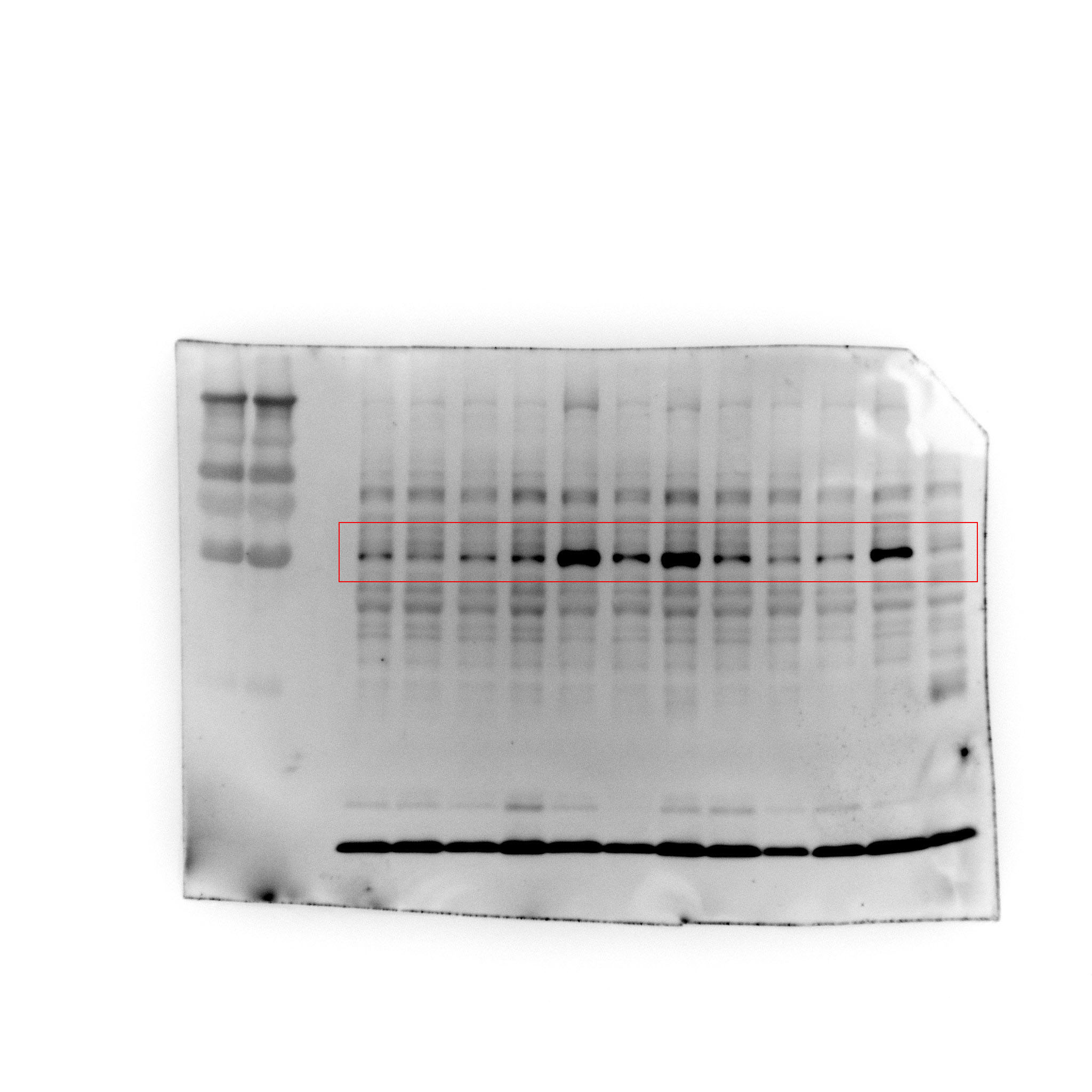
**

**Figure 2A:** Oxy-CaMK


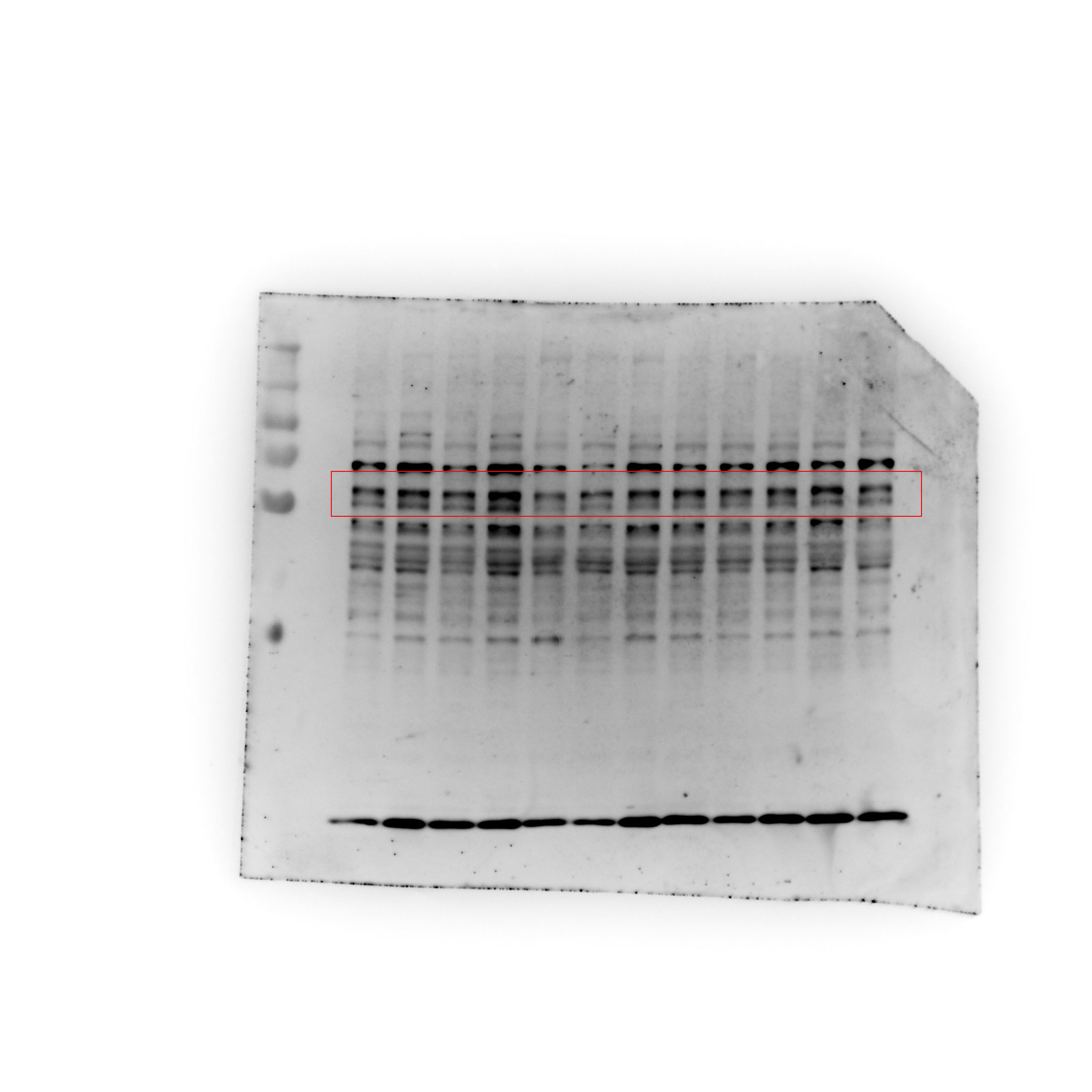


**Figure 2A:** Total CaMK

**
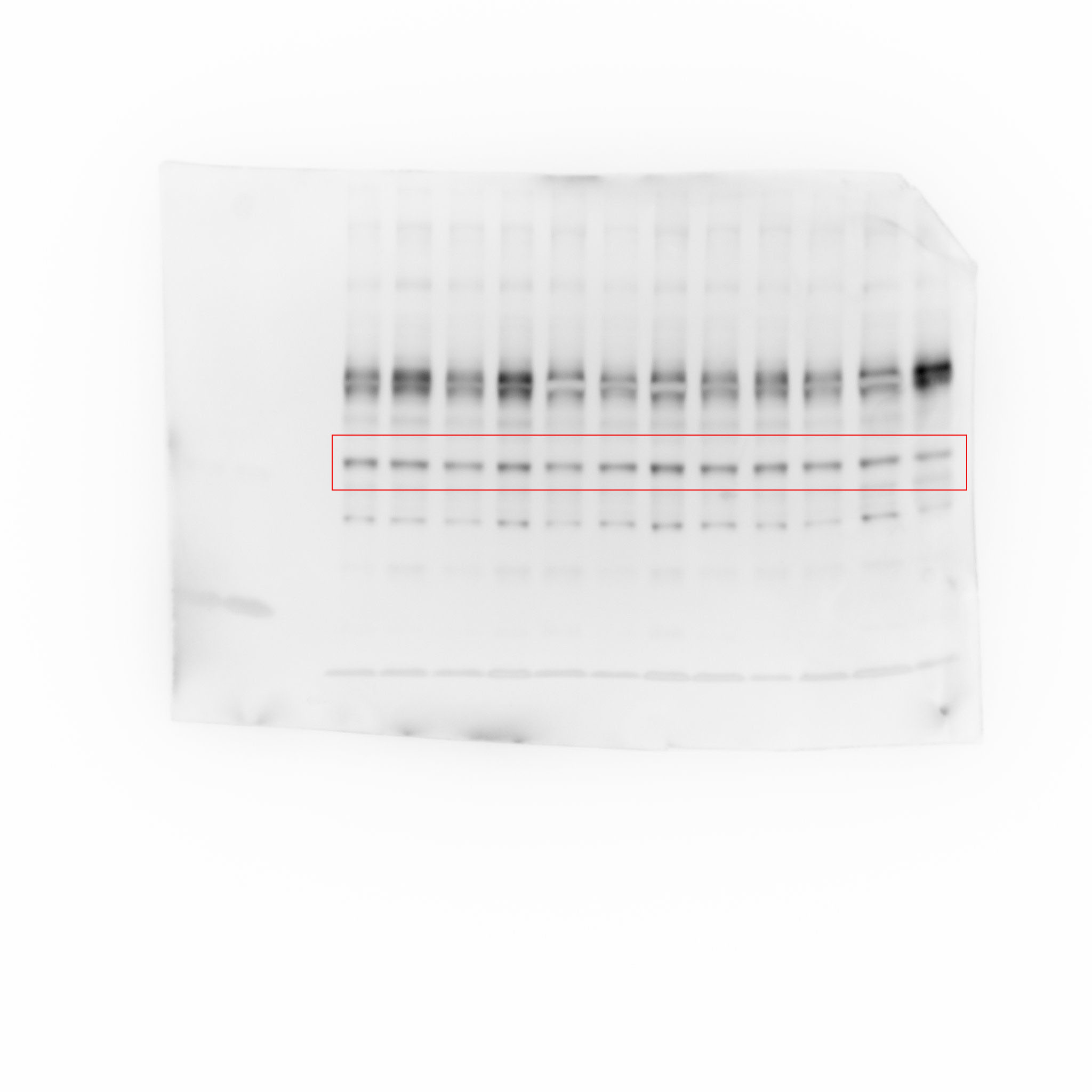
**

**Figure 2A:** Ponceau CaMK

**
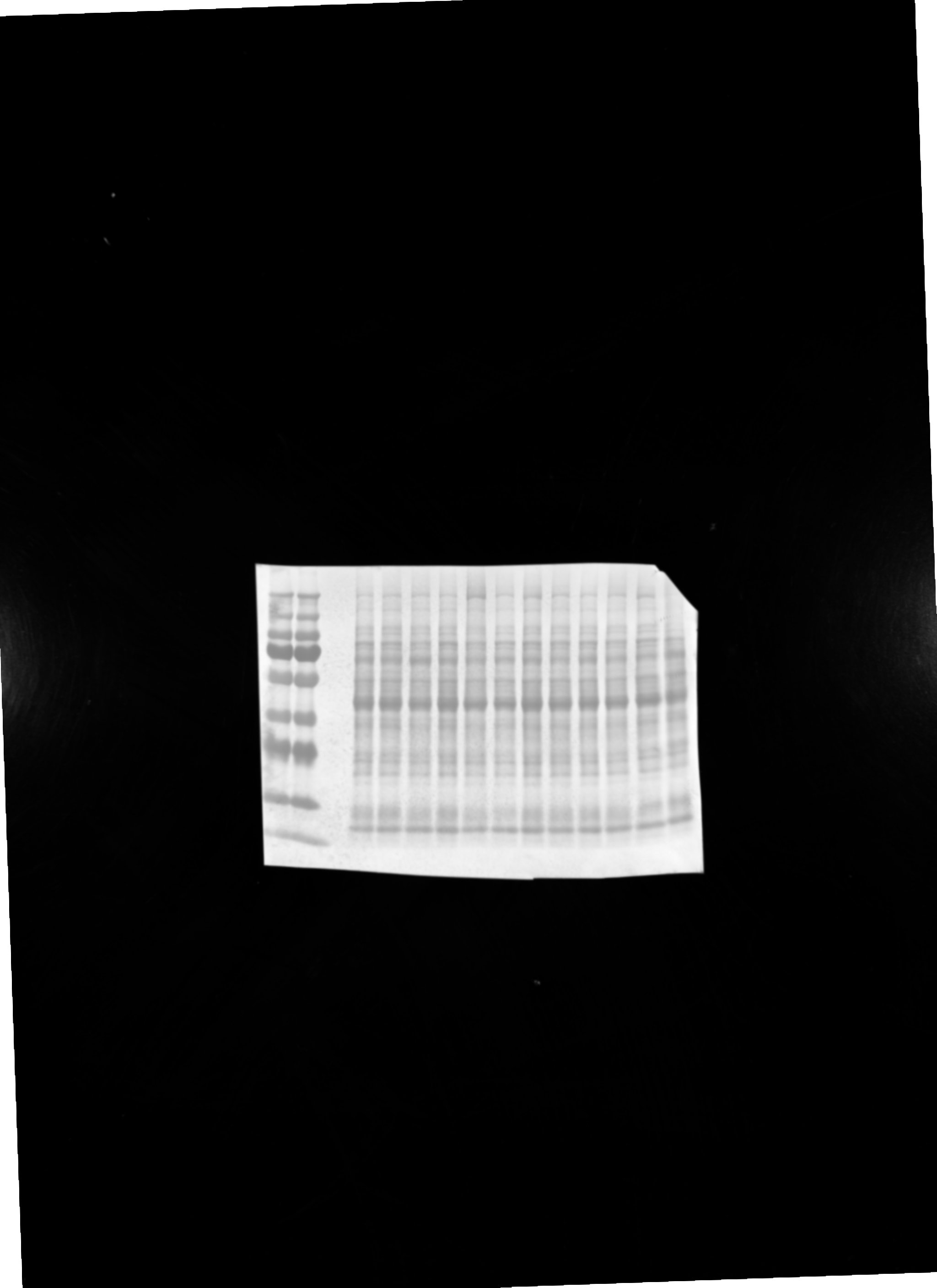
**

**Figure 2G:** Oxy RyR2

**
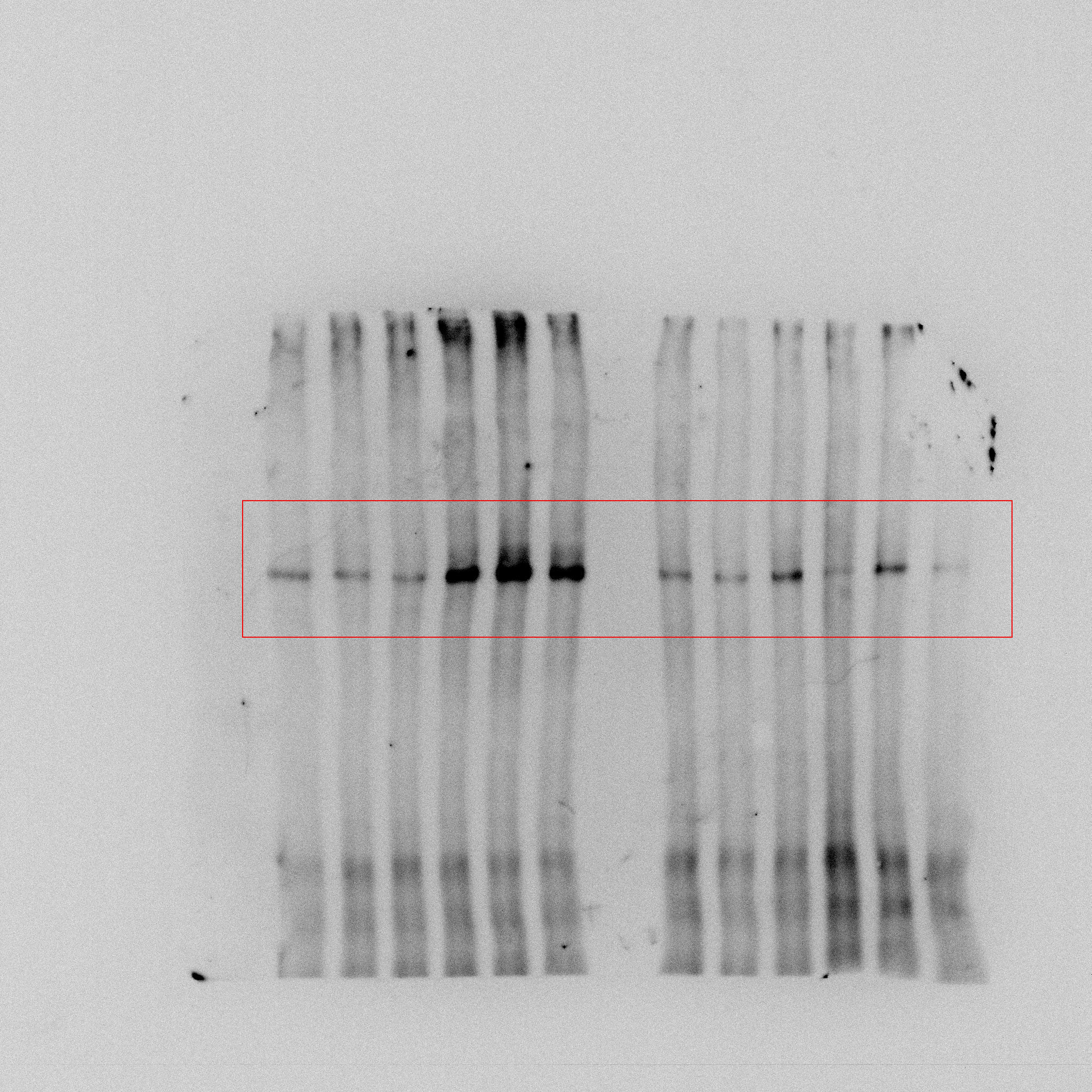
**

**Figure 2G:** Phospho RyR2

**
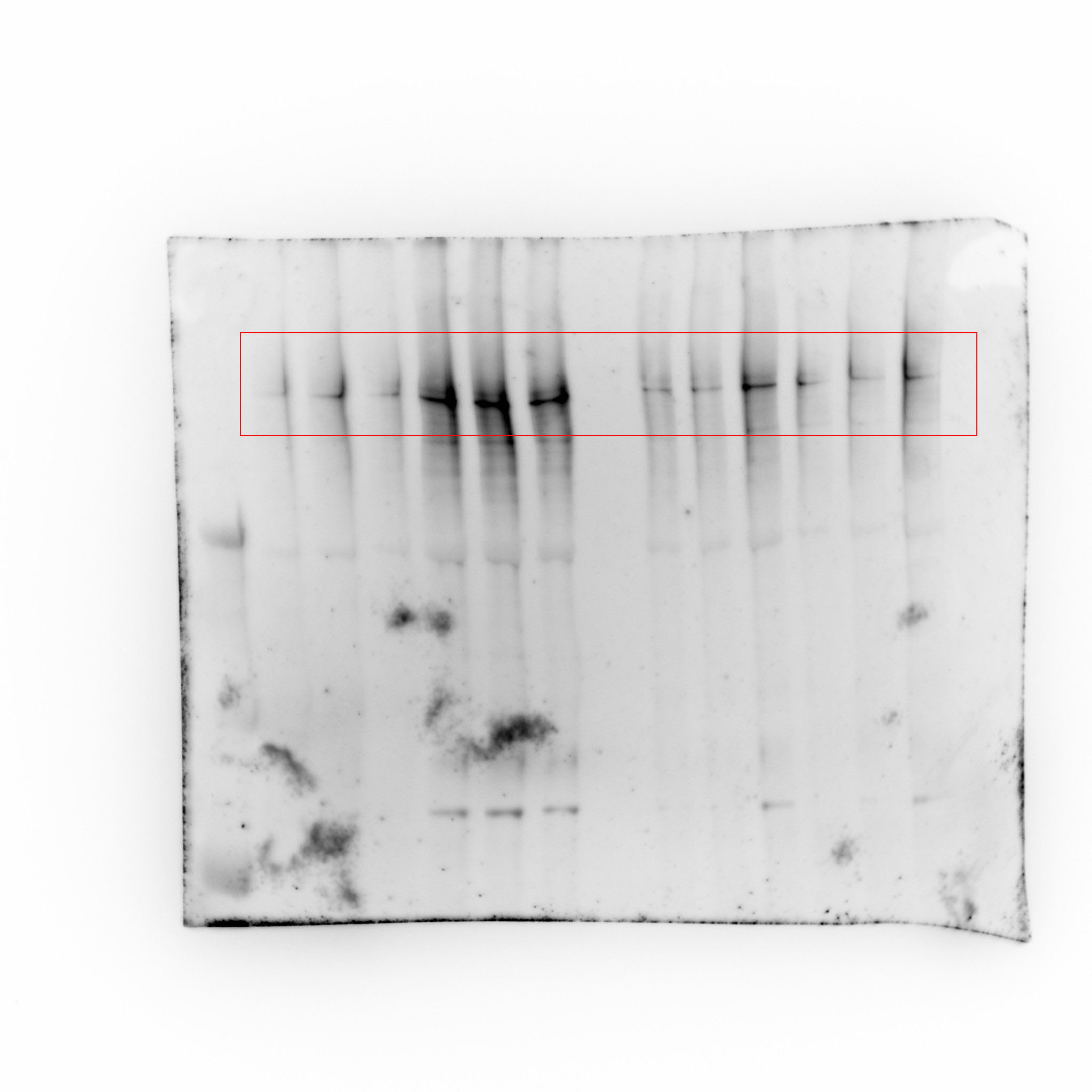
**

**Figure 2G:** total RyR2

**
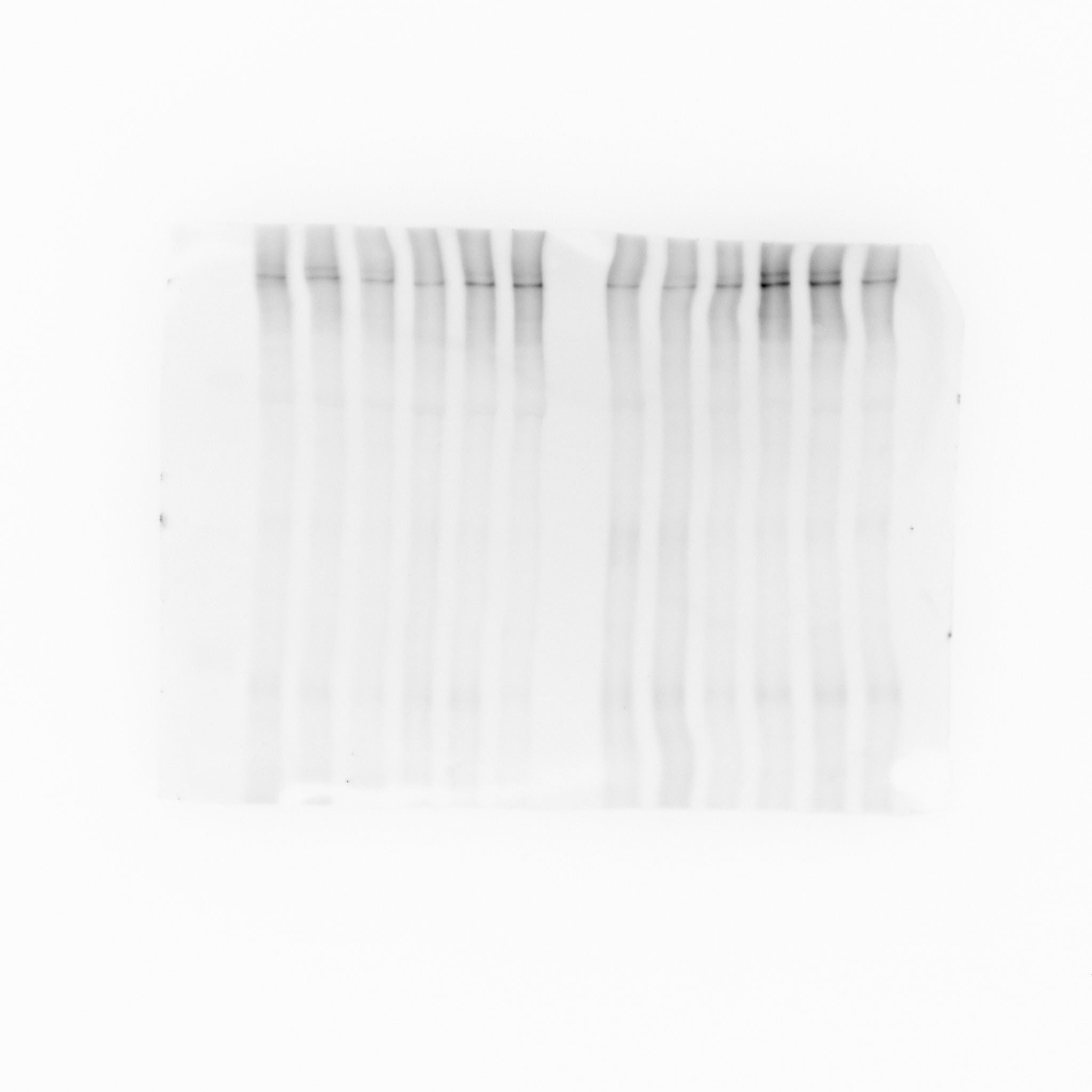
**

**Figure 3A: Kv1.5.** For this blot and the next two, an older lysate was loaded next to the ladder to determine if the amount of protein loaded was similar to prior work.

**
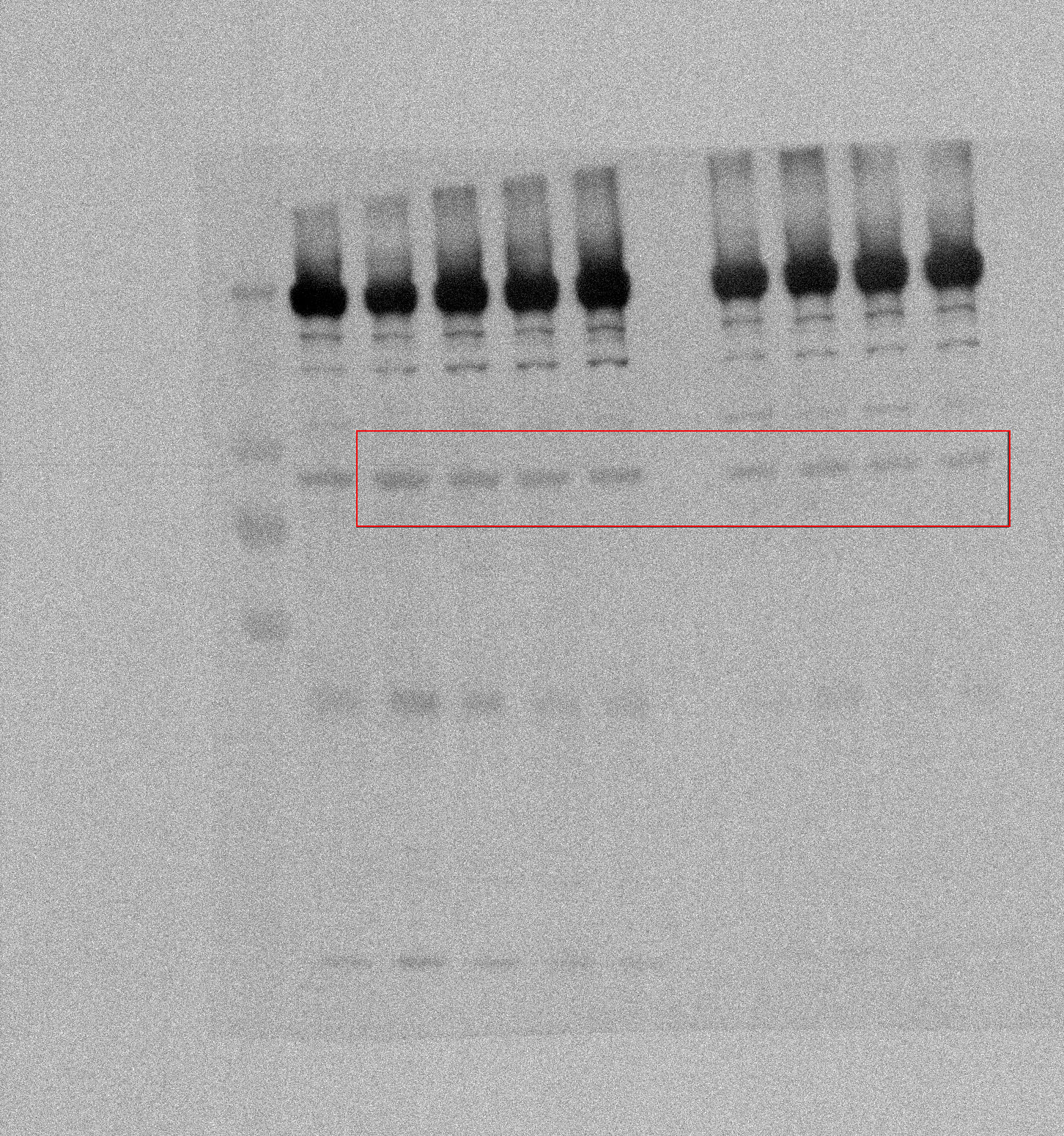
**

**Figure 3A: Ponceau**

**
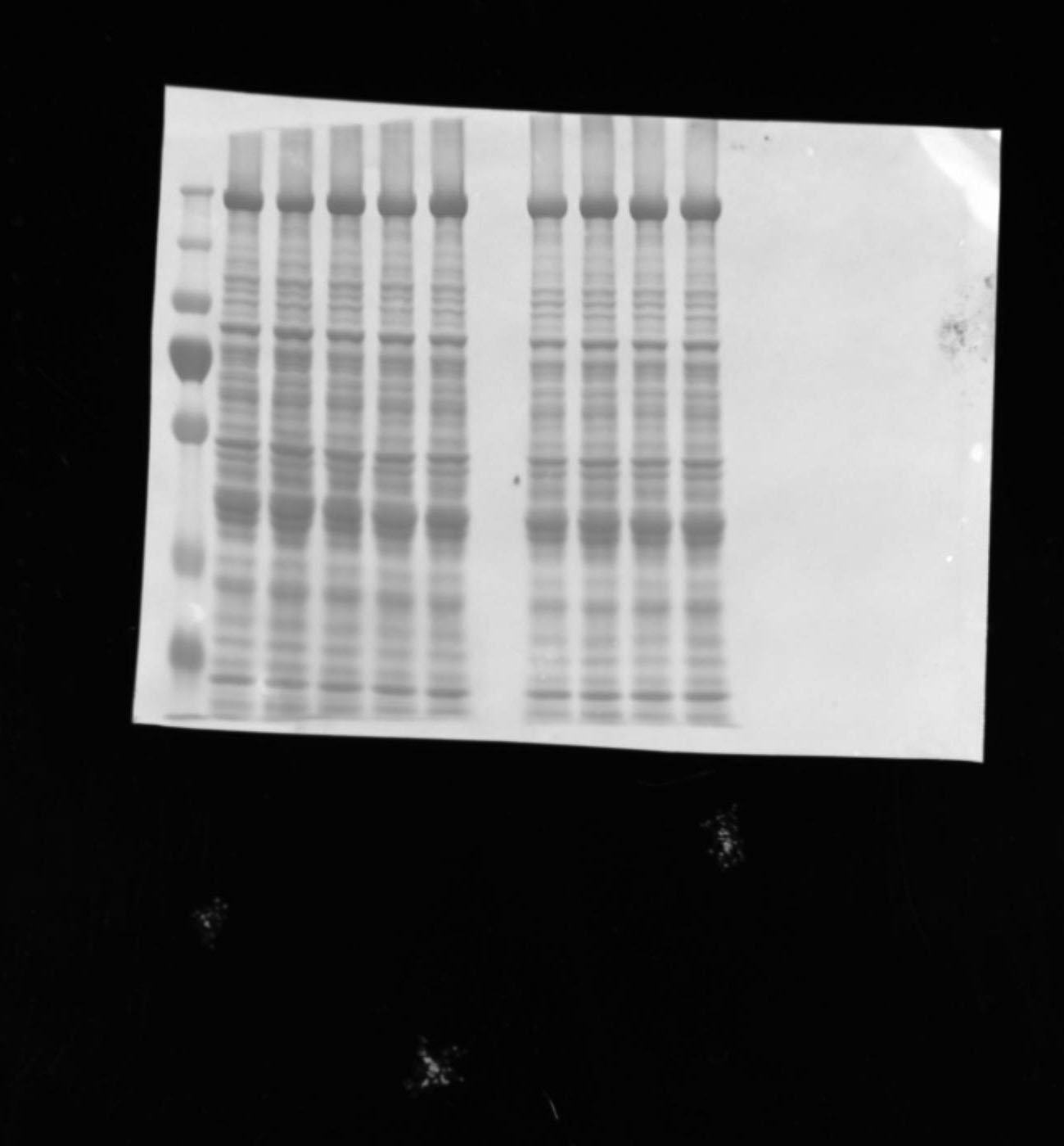
**

**Figure 3B: Kv2.1**

**
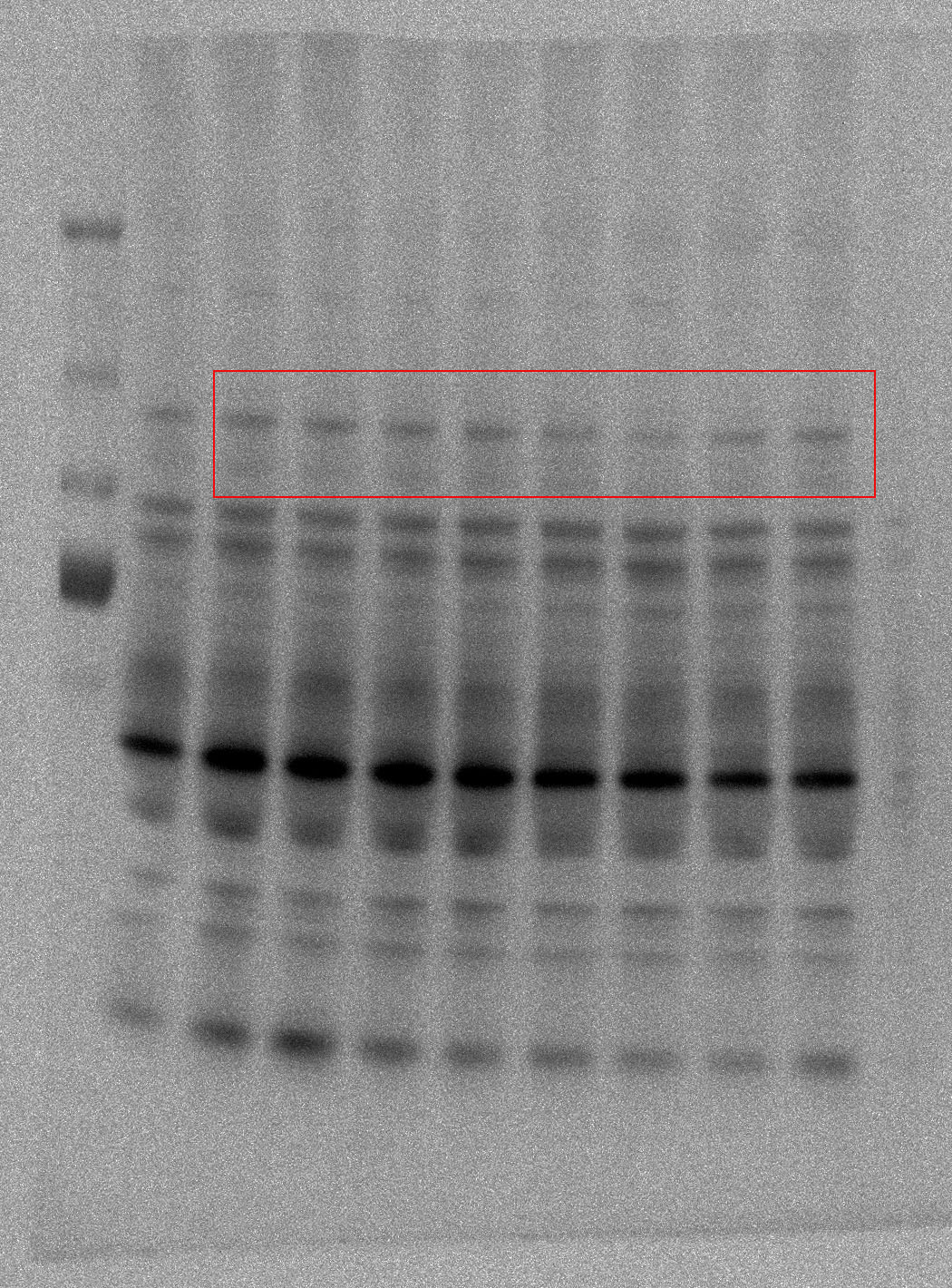
**

**Figure 3B: Ponceau**

**
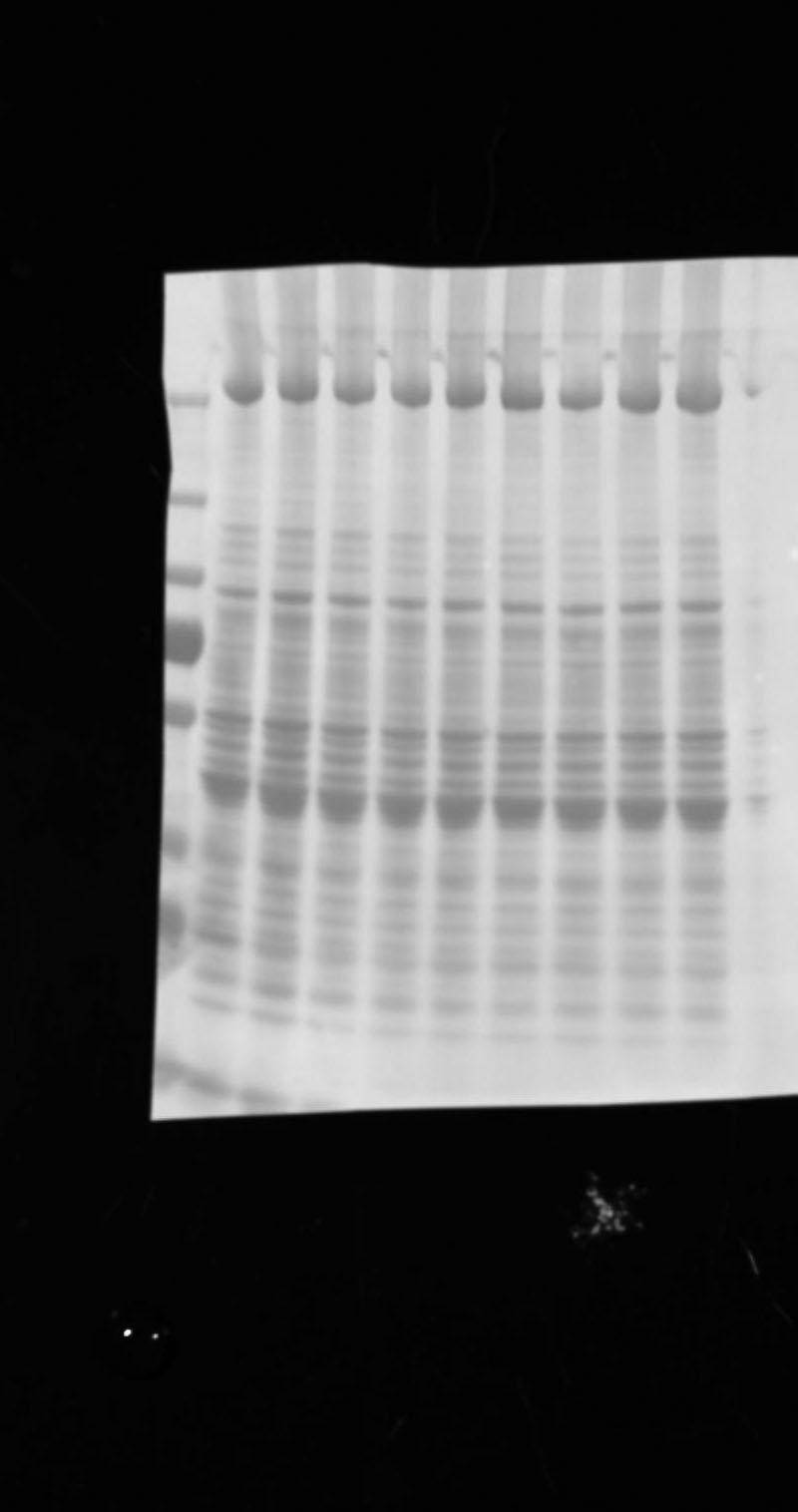
**

**Figure 3C: Kv4.2**

**
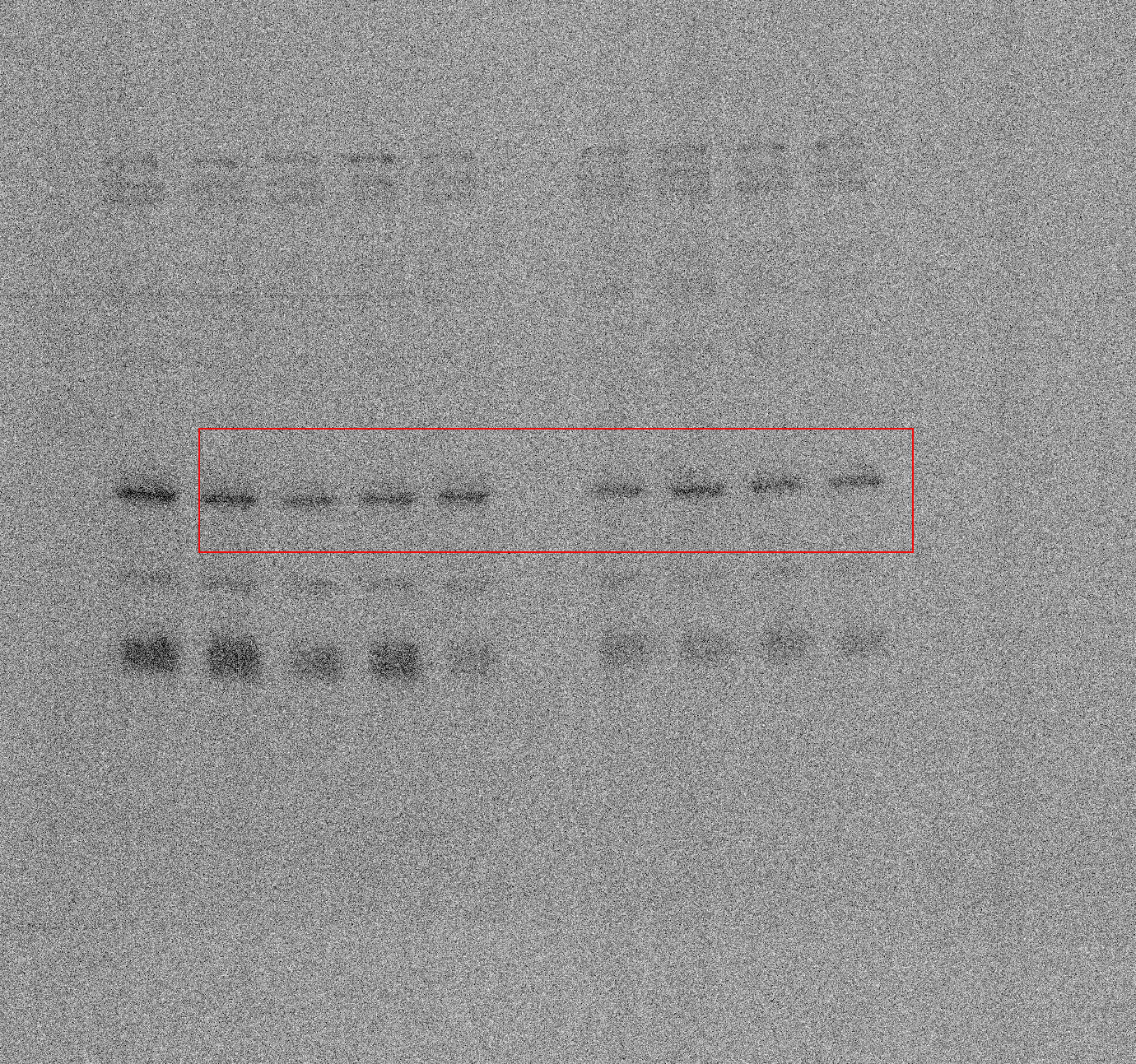
**

**Figure 3C: Ponceau**

**
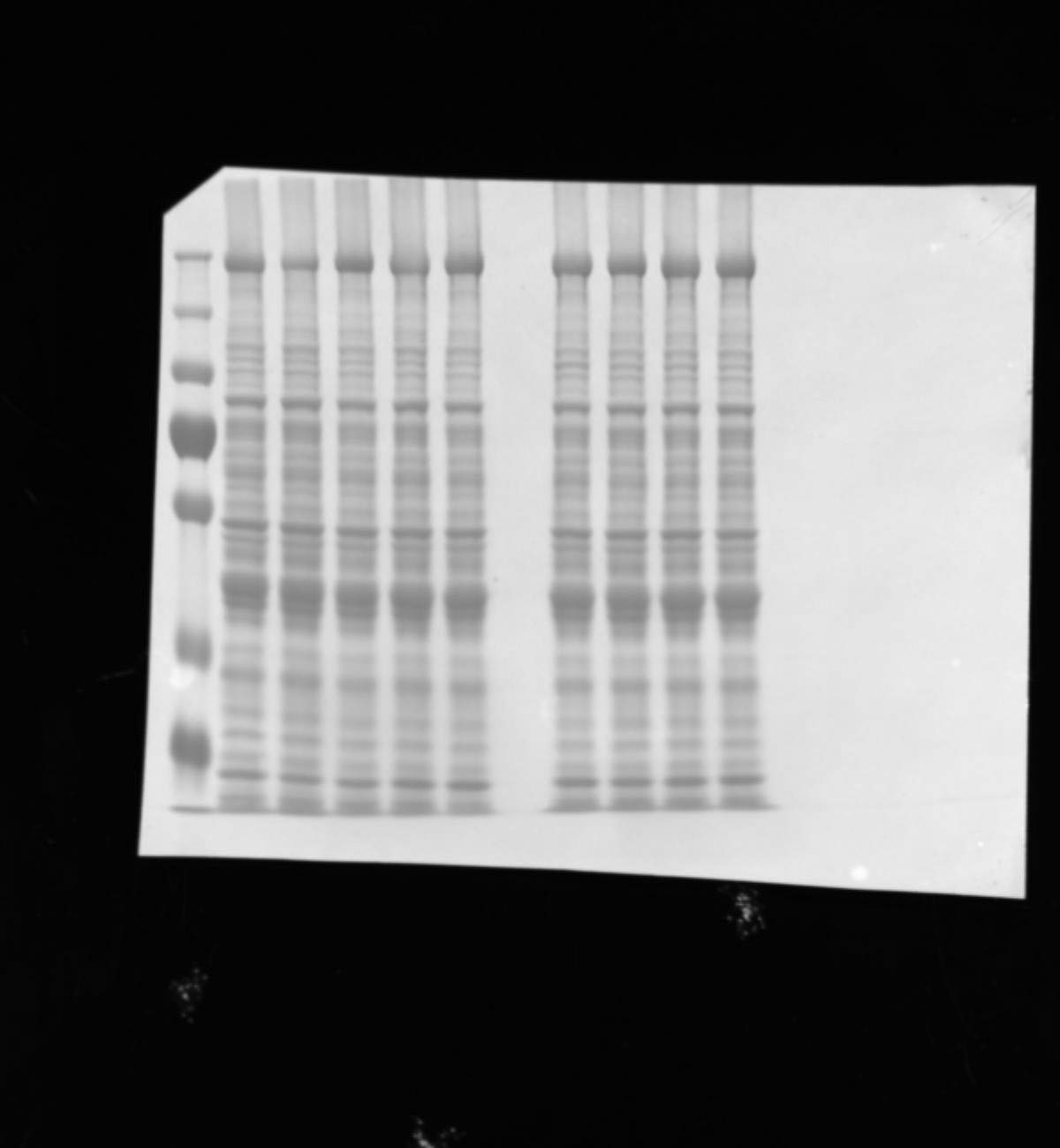
**

**Figure 3D: Kv1.5**


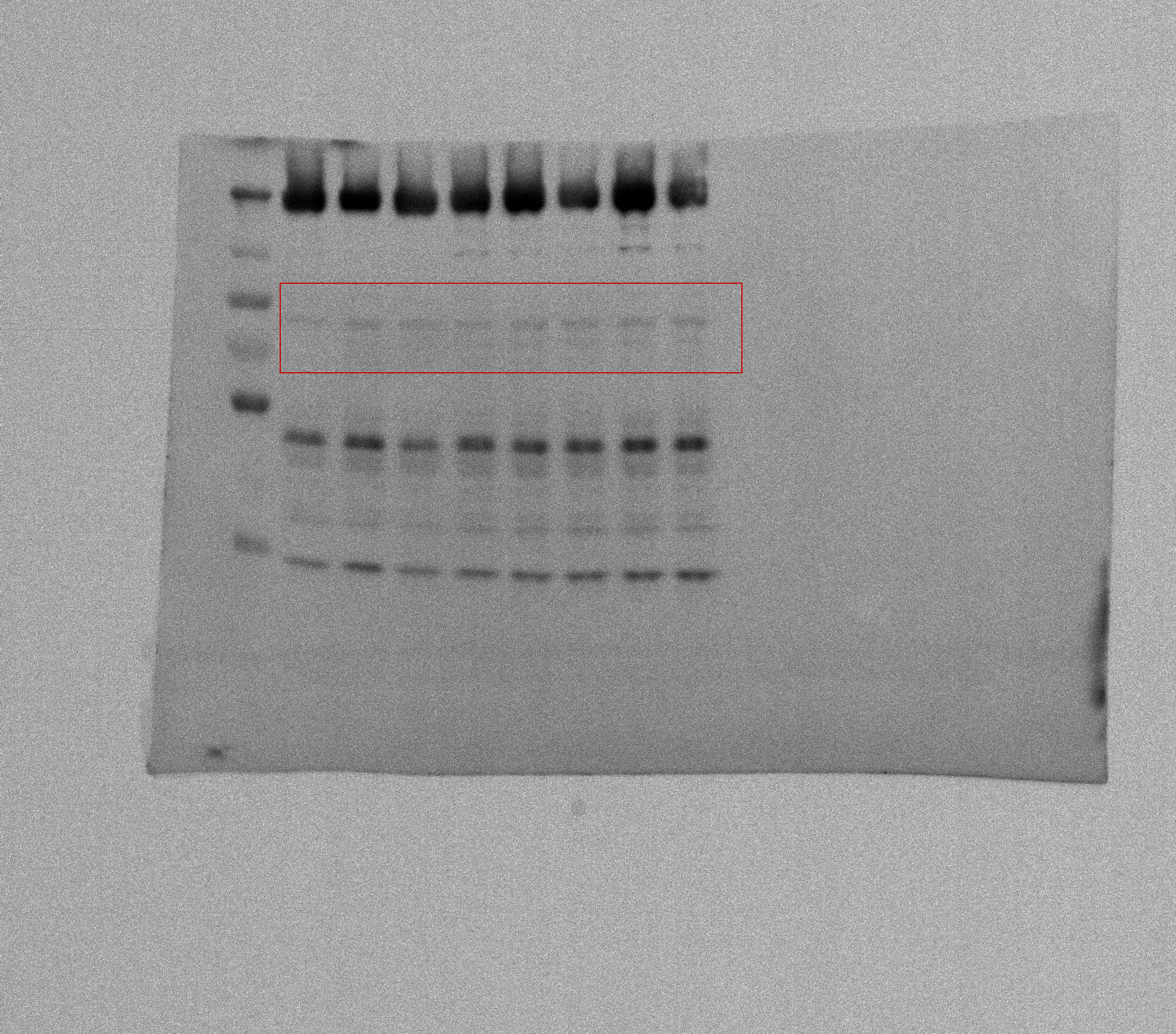


**Figure 3D: Ponceau**


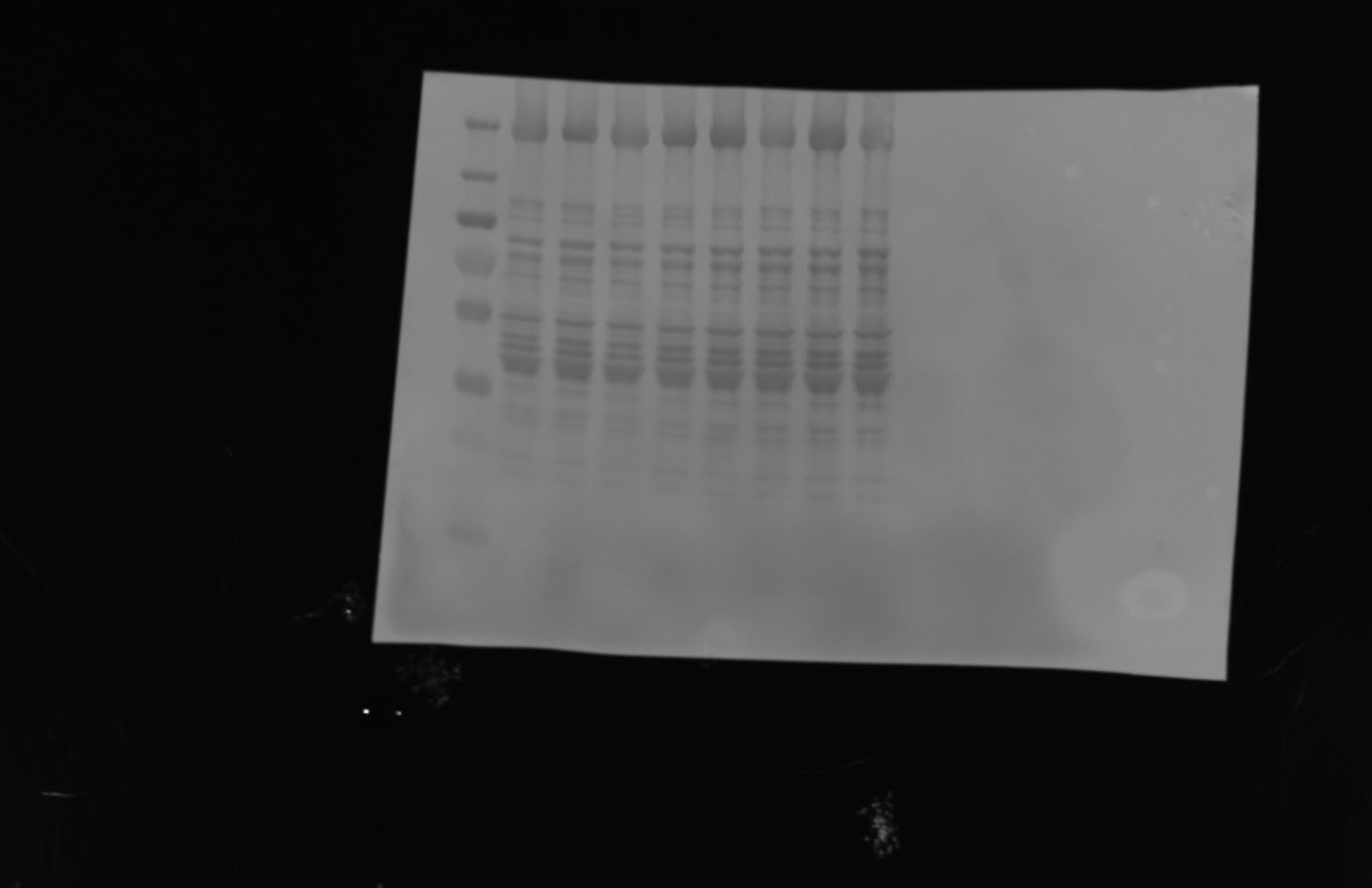


**Supplement Figure 1: MCU western blot, control mice**


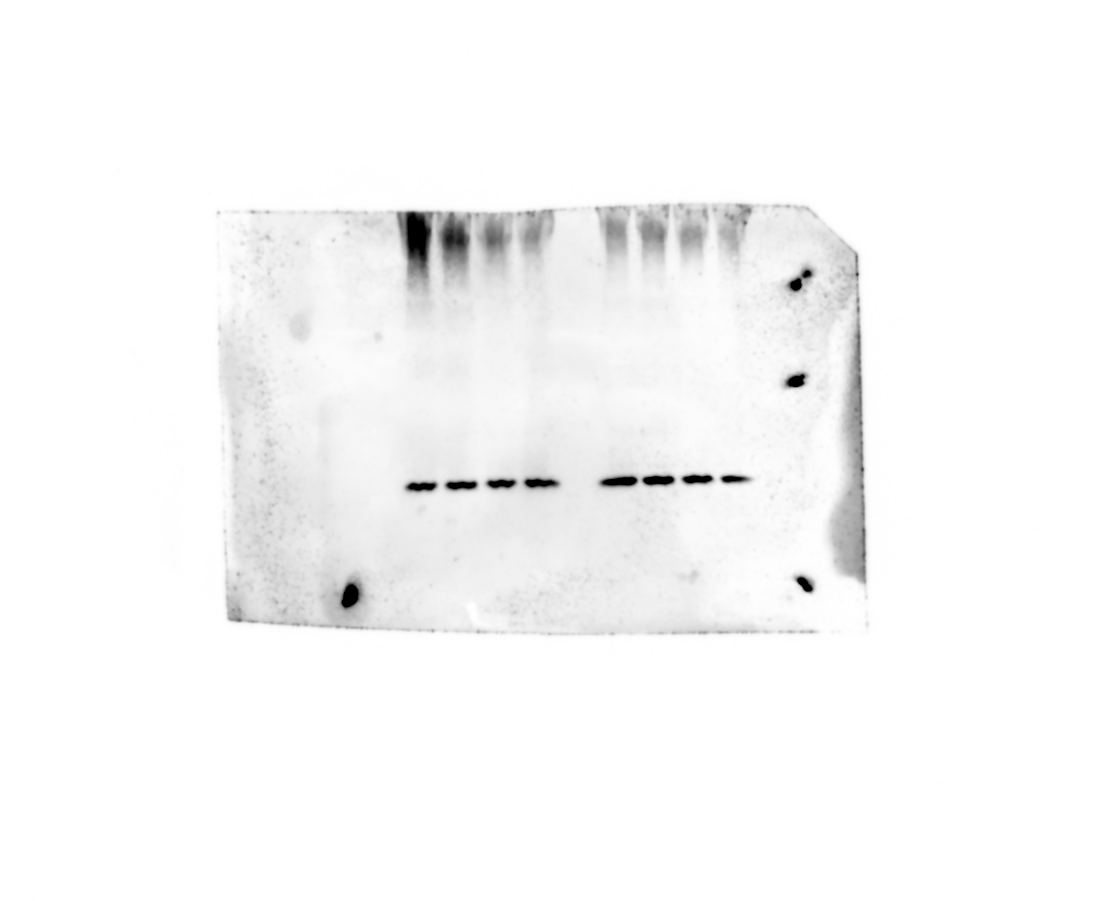


**Supplement Figure 1: Ponceau**


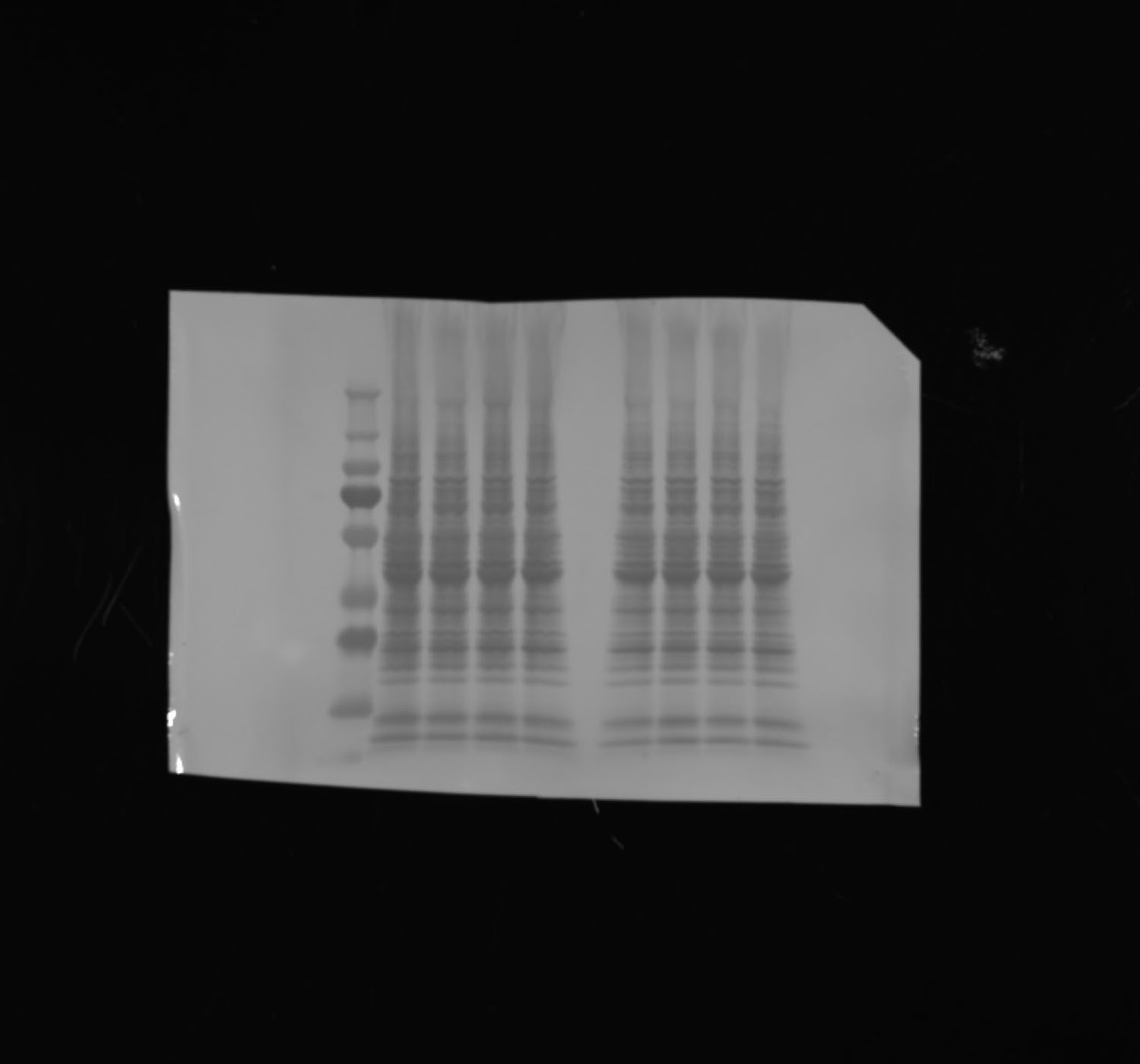


**Supplement Figure 3: MCU western blot, control mice and CaMKi mice**


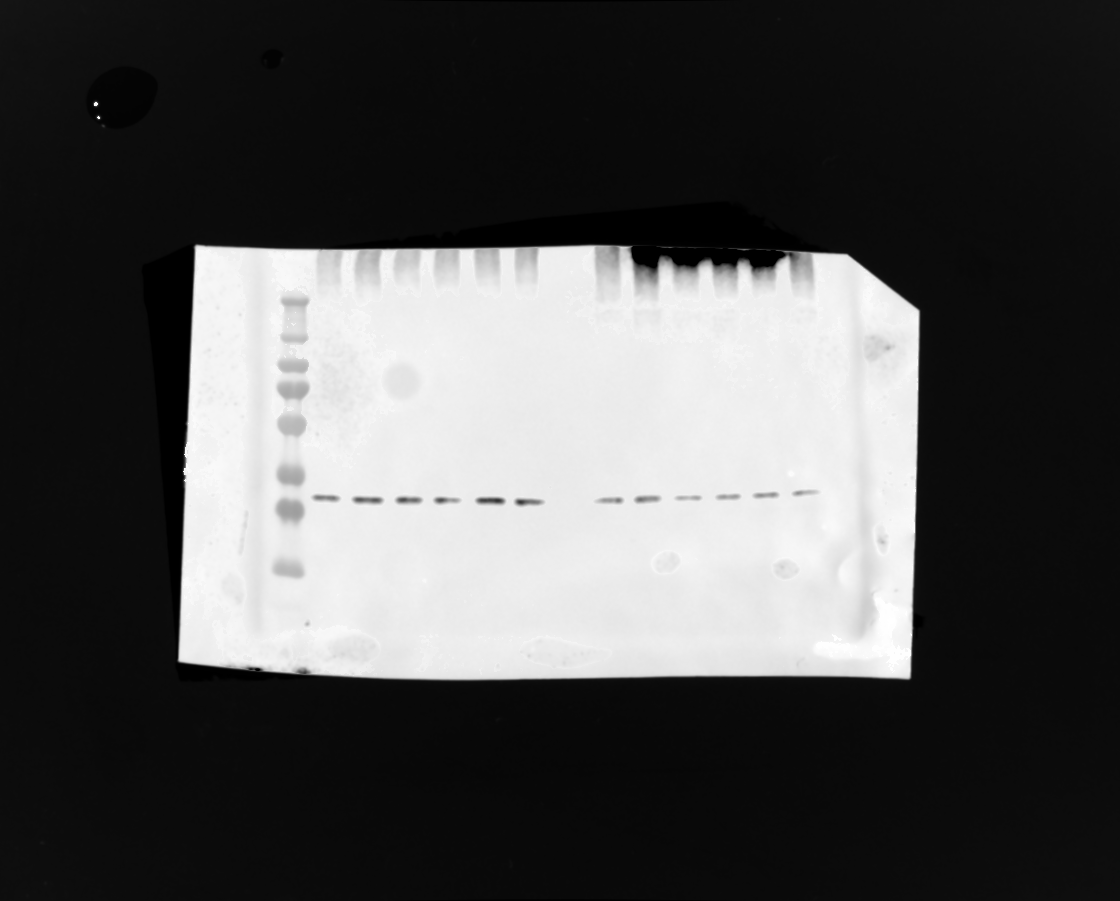


**Supplement Figure 3: Ponceau**


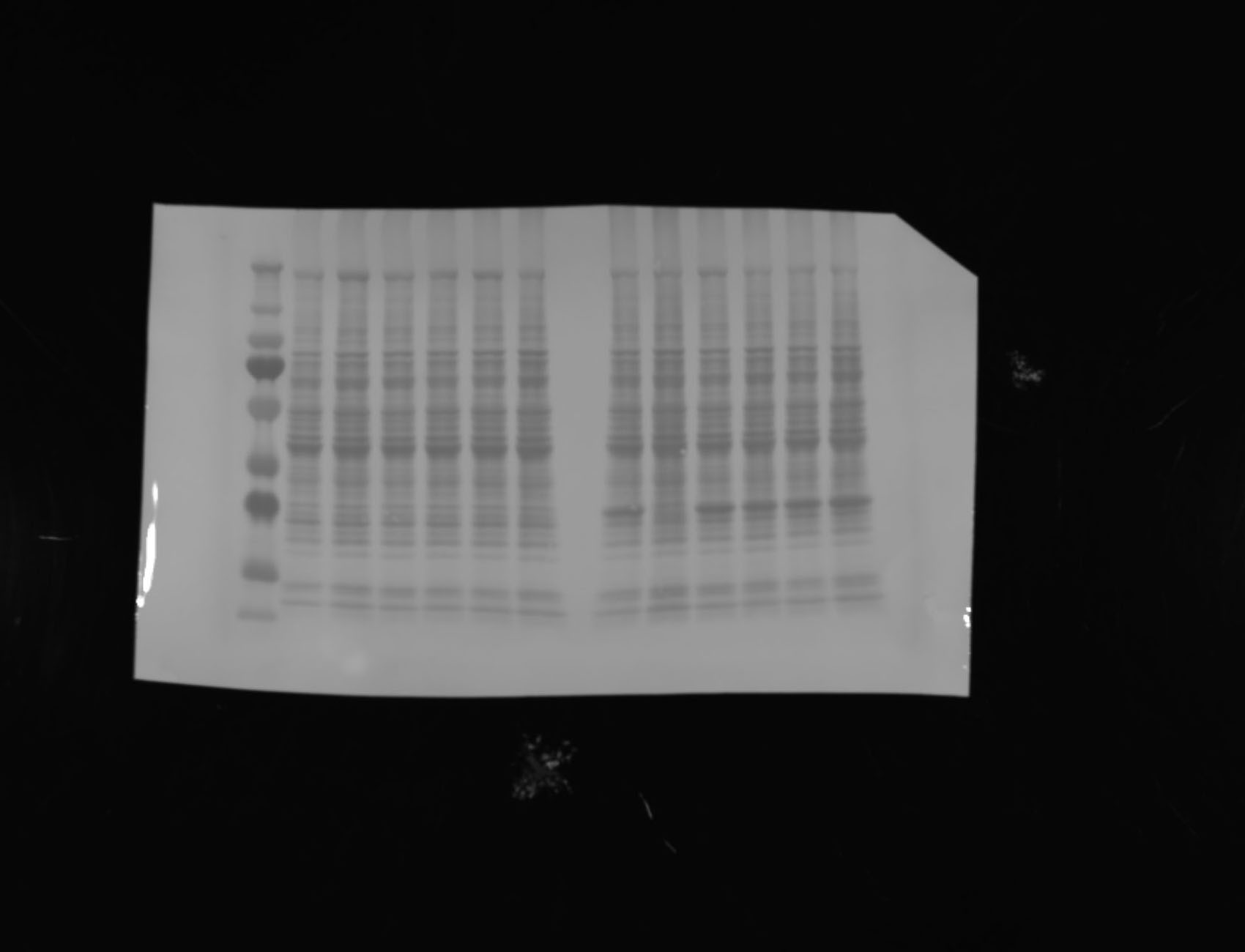

Supplement: Supplementary file 1 — Supplementary Information. [file 41598_2021_97449_MOESM1_ESM.docx]
